# Metal–Organic Frameworks Built with Selenophene-Based Trimers for Perfluoroalkyl Substances Removal from Drinking Water

**DOI:** 10.1021/acs.chemmater.6c00594

**Published:** 2026-07-10

**Authors:** Giulio Bicchierai, Andrea Trifoglio, Laura Favaretto, Daniele Evandri, Sara Khaliha, Ilse Manet, Lidia Armelao, Gregorio Bottaro, Anna Mauri, Simona Galli, Giuliano Giambastiani, Manuela Melucci, Andrea Rossin

**Affiliations:** † Istituto di Chimica dei Composti Organometallici, CNR ICCOM, via Madonna del Piano 10, Sesto Fiorentino, 50019 Firenze, Italy; ‡ Istituto per la Sintesi Organica e la Fotoreattività, CNR ISOF, Via P. Gobetti 101, I-40129 Bologna, Italy; § Dipartimento di Chimica ‘G. Ciamician’, Alma Mater Studiorum—Università di, Via Selmi 2, 40126 Bologna, Italy; ∥ Dipartimento di Scienze Chimiche (DiSC), Università di Padova, via F. Marzolo 1, 35131 Padova, Italy; ⊥ Dipartimento di Scienze Chimiche e Tecnologie dei Materiali (DSCTM), Consiglio Nazionale delle Ricerche (CNR), Piazzale A. Moro 7, 00185 Roma, Italy; # Istituto di Chimica della Materia Condensata e di Tecnologie per l’Energia, CNR-ICMATE, Via F. Marzolo 1, 35131 Padova, Italy; ¶ Consorzio Interuniversitario per la Scienza e Tecnologia dei Materiali (INSTM), Via Giusti 9, 50121 Firenze, Italy; ∇ Dipartimento di Scienza e Alta Tecnologia, Università degli Studi dell’Insubria, Via Valleggio 9, 22100 Como, Italy; ○ Dipartimento di Chimica “Ugo Schiff”, Università di Firenze, via della Lastruccia 3-13, Sesto Fiorentino, 50019 Firenze, Italy

## Abstract

Per- and polyfluoroalkyl substances (PFAS) are persistent organic pollutants that have attracted growing concern owing to their widespread presence in drinking water and limited removal by conventional remediation technologies. This challenge has driven increasing interest in porous materials capable of selectively removing PFAS under environmentally relevant conditions. Among these, zirconium-based metal–organic frameworks (MOFs) have shown great potential for water-related applications due to their chemical and hydrolytic stability. In this work, we report the design, synthesis, and characterization of two UiO-68-type mixed-linker MOFs, **UiO-68-SSeS** and **UiO-68-TzSeTz**, incorporating selenophene-based heteroaromatic dicarboxylate linkers through a solvent-assisted linker exchange strategy. The heterocyclic linkers 5,5′-(selenophene-2,5-diyl)­bis­(thiophene-2-carboxylate) (H_2_SSeS) and 2,2′-(selenophene-2,5-diyl)­bis­(thiazole-5-carboxylate) (H_2_TzSeTz) were successfully embedded within UiO-68 while preserving its **fcu** topology. The performance of the two MOFs as sorbents toward PFAS in tap water was evaluated under environmentally relevant conditions using a mixture of PFAS differing in chain length and functional group. Both materials display a clear selectivity toward long-chain PFAS, with **UiO-68-SSeS** showing particularly high removal efficiency for perfluoroundecanoic acid (PFUnDA). These results demonstrate that rational linker design represents an effective approach to modulate the adsorption behavior of UiO-type MOFs, opening future perspectives for functional materials in water remediation.

## Graphical abstract

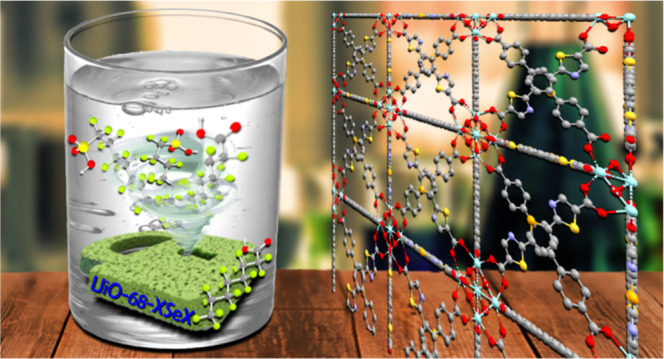

## 1 Introduction

The increasing presence of per- and polyfluoroalkyl substances (PFAS) in water resources has become a critical global concern due to their environmental persistence, bioaccumulation potential, and adverse effects on ecosystems and human health.^1,2^ PFAS are frequently detected in raw water sources intended for drinking water treatment, often at concentrations that challenge conventional purification processes.^3^ The chemical complexity and exceptional stability of these compounds pose significant challenges to existing drinking water treatment technologies, calling for improved monitoring, mitigation, and remediation strategies.^4^ PFAS are characterized by strong carbon–fluorine bonds exhibiting exceptional thermal and chemical stability, which underpins their widespread industrial and consumer applications, including firefighting foams, nonstick coatings, textiles, and food packaging.^5–8^ However, these same properties render PFAS highly resistant to environmental degradation, making them the so-called “forever chemicals”. Conventional drinking water treatment plants are not specifically designed to address PFAS contamination, and many treatments provide only limited removal.^9^ As a result, the presence of PFAS in drinking water represents a crucial challenge for water potabilization facilities and regulators, with significant implications for long-term human exposure.^10^ In this context, pollutants adsorption by functional materials like metal–organic frameworks (MOFs) have gained growing attention as a promising approach for PFAS mitigation. MOFs are a class of porous crystalline materials composed of metal nodes coordinated to organic linkers, forming highly tunable three-dimensional networks. Their defining features (ultrahigh surface area, adjustable pore size, and chemical functionality) make them particularly attractive for applications in environmental remediation.^11–14^ MOFs relevance in contemporary chemistry is further emphasized by the assignment of the Nobel Prize in Chemistry 2025 to Susumu Kitagawa, Richard Robson, and Omar Yaghi for their pioneering work in this area.^15^ In recent years, MOFs have gained increasing attention as adsorbent materials for the removal of persistent pollutants, including PFAS, from aqueous environments.^16^ Their uniquely tailored pore environment allows MOFs to interact selectively with PFAS through mechanisms such as hydrophobic interactions, electrostatic attractions, and π–π stacking, enhancing affinity for PFAS fluorinated chains and thereby improving adsorption capacity and selectivity even at trace concentrations typically found in contaminated waters. Aromatic heterocycles are particularly attractive as linkers or functional groups in MOFs, as their heteroatoms tune hydrophobicity and influence the adsorption affinity toward organic pollutants. Thiophenes and related heteroaromatic oligomers (i.e., thiazoles and selenophenes) have been widely investigated in the fields of photonics and organic electronics, especially in their oligomeric form, due to their optical and electronic properties.^17–19^ By tailoring their molecular architecture, in particular the extent of π-conjugation and the nature of substituents on the aromatic backbone, their chemical and photophysical behavior can be finely tuned, enabling their exploitation across a broad range of applications including water remediation. Recently, some of us have studied oligothiophene-based systems as photocatalysts for the degradation of organic contaminants in water, demonstrating the versatility and water processability of this class of materials.^20^ In addition, fluorescent thiophene-containing systems have recently shown photochemical responses upon interaction with PFAS, resulting in fluorescence changes or absorption variations, enabling their application as sensors for PFAS detection in water.^21,22^ Selenophene, thiophene, and thiazole are also widely employed in materials science and coordination chemistry because of their structural properties. Thiophene is a five-membered sulfur-containing ring known for its planarity, aromaticity, and electron-rich character, which facilitate π–π stacking interactions. Selenophene, the selenium analogue of thiophene, exhibits similar aromaticity but contains a heteroatom with a larger atomic radius and higher polarizability, enhancing noncovalent interactions and potentially improving the adsorption properties of derived materials. Thiazole is a five-membered ring containing both nitrogen and sulfur, combining electron-donating and electron-withdrawing character that can modulate the electronic density in MOFs. An important feature to be taken into consideration for applications in aqueous environments is water stability. Zirconium-based MOFs, such as the prototypical UiO-6*x* family,^23^ are widely recognized for their exceptional stability in aqueous environments compared to many other MOF classes. This robustness arises from the 12-connectivity of their [Zr_6_O_4_(OH)_4_]^12+^ clusters, which confers notable resistance to hydrolysis and structural collapse, making them suitable for water-related applications. In recent years, some of us have reported on a number of Zr^IV^ MOFs containing heterocyclic linkers for the sensing and/or adsorption of the anti-inflammatory drugs Diclofenac Sodium or Ibuprofen Sodium in water.^24–27^ Following this direction and combining the research interests of our teams, in this work, we present two mixed-linker Zirconium MOFs, **UiO-68-SSeS** and **UiO-68-TzSeTz**, derived from UiO-68 [the fully carbocyclic MOF built with the commercially available (*p*-terphenyl)-4,4′′-dicarboxylic acid][Bibr ref23] and functionalized with the designed heterocyclic selenophene-based linkers whose synthesis is described here for the first time (Scheme 1). PFAS adsorption capability in tap water of these materials is herein described.

**Scheme 1:**
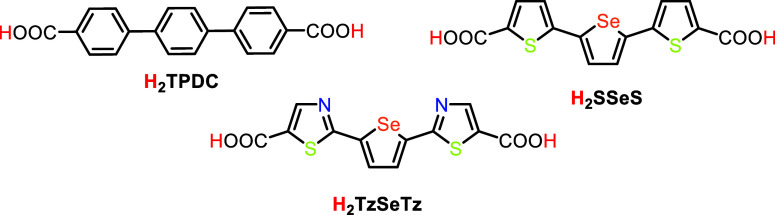
Ditopic Linkers Used in This Study for the Construction of the UiO-68-Type Zr^IV^ MIXMOFs **UiO-68-SSeS** and **UiO-68-TzSeTz**: (*p*-Terphenyl)-4,4″-dicarboxylic Acid (H_2_TPDC), 5,5′-(Selenophene-2,5-diyl)­bis­(thiophene-2-carboxylic acid) (H_2_SSeS), and 2,2′-(Selenophene-2,5-diyl)­bis­(thiazole-5-carboxylic acid) (H_2_TzSeTz)

## 2 Experimental Section

### 2.1 Materials and Methods

Zirconium chloride (ZrCl_4_, Strem Chemicals Ltd.), zirconium dichloride oxide hydrate (ZrOCl_2_·*x*H_2_O, Strem Chemicals Ltd.), trifluoroacetic acid (CF_3_COOH, TFA, Sigma-Aldrich), (*p*-terphenyl)-4,4″-dicarboxylic acid (H_2_TPDC, BLD Pharmatech Ltd.), *N*,*N*-dimethylformamide (DMF, Sigma-Aldrich), 2,5-dibromoselenophene (TCI), 5-bromothiophene-2-carboxylic acid (Sigma-Aldrich), tributylchlorostannane (Sigma-Aldrich), *n*-butyllithium (Sigma-Aldrich), 2-bromothiazole-5-carboxylic acid (Sigma-Aldrich), *tert*-butanol (^*t*^BuOH, Sigma-Aldrich), dimethylaminopyridine (DMAP, Sigma-Aldrich), pyridine (py, Sigma-Aldrich), di *tert*-butyl dicarbonate (BOC_2_O, Sigma-Aldrich), *N*-(3-(dimethylamino)­propyl)-*N*′-ethylcarbodiimide (EDC, Sigma-Aldrich), tetrakis­(triphenylphosphine)­palladium(0) ([Pd­(PPh_3_)_4_], Sigma-Aldrich), and toluene (Tol, Sigma-Aldrich) were purchased from commercial vendors and used as received. UiO-68 was prepared starting from ZrCl_4_, H_2_TPDC, and TFA as a crystal modulator, following the literature procedure reported by Lin et al. in 2016.^28^ PFAS standard MIXs were purchased from Agilent Technologies (Santa Clara, CA, US). The experiments on PFAS were carried out by using polypropylene vials. ^1^H and ^13^C NMR spectra related to H_2_SSeS and H_2_TzSeTz characterization were recorded with a Varian Mercury 400 spectrometer (400 MHz for ^1^H NMR and 100 MHz for ^13^C NMR spectra). In the following, the chemical shifts (δ) are reported in ppm (parts per million) referred to the signals of the residual solvents (^1^H CHCl_3_ δ_Η_ = 7.26 ppm and DMSO δ_Η_ = 2.48 ppm; ^13^C CHCl_3_ δ_C_ = 77.0 ppm, and DMSO δ_C_ = 40.0 ppm). Coupling constants (*J*) are reported in Hz, and multiplicities are named by the following abbreviations: singlet (s), doublet (d), and multiplet (m). Mass spectrometry (MS) spectra related to H_2_SSeS and H_2_TzSeTz were acquired by electron impact ionization at 70 eV on a Thermo Scientific Trace 1300 GC, ISQ 7610 Single Quadrupole MS. The sample was introduced in the ion source region via a direct exposure probe. Melting Point (M.P.) was recorded with a Buchi 510 instrument with silicone oil. ^1^H, ^13^C, and ^19^F NMR spectra of the digested MOFs were recorded on BRUKER AVANCE 400/600 MHz spectrometers. Chemical shifts (δ) are reported in parts per million (ppm) downfield of tetramethylsilane (TMS, ^1^H, ^13^C) or trichlorofluoromethane (CFCl_3_, ^19^F) and calibrated against the residual protiated solvent resonance. Linkers quantification via ^1^H NMR analysis was performed by digesting the solid (*ca*. 10 mg) in 0.7 mL of a D_2_SO_4_/D_2_O/DMSO-*d*_6_ mixture heated at *T* = 353 K for 2 h. Subsequently, the ^1^H spectrum was collected adopting the following conditions: acquisition time 20 min, relaxation delay τ = 16 s, and 216 scans. FT-IR spectra (KBr pellets) were recorded on a PerkinElmer Spectrum BX Series FTIR spectrometer, in the 4000–400 cm^–1^ range, with a 2 cm^–1^ resolution. Thermogravimetric analyses (TG-DTG) were performed under a N_2_ flow (100 mL min^–1^) at a heating rate of 5 K min^–1^ with an EXSTAR Thermogravimetric Analyzer Seiko 6200. The latter was coupled with a ThermoStar GSD 301T mass spectrometer for mass analysis of the volatile species. The elemental analyses were performed on thermally activated batches of the two MIXMOFs using a Thermo FlashEA 1112 Series CHNS–O elemental analyzer with an accepted tolerance of ±2% on carbon (C), hydrogen (H), nitrogen (N), and sulfur (S). The nature and purity of all the batches employed for the functional characterization were assessed through powder X-ray diffraction (PXRD). PXRD qualitative measurements were carried out in the 2.0–50.0° 2θ region with a Panalytical X’PERT PRO diffractometer equipped with a Ni filter in the diffracted beam, a PIXcel^©^ solid-state detector, and a sealed X-ray tube (Cu Kα, λ = 1.5418 Å). Slits were used on both the incident (Soller slits aperture: 0.25°; divergence slit aperture: 0.5°) and the diffracted (antiscatter slit aperture: 7.5 mm) beam. The generator was operated at 40 kV and 40 mA. Measurements were carried out in reflection mode, using a spinning circular sample holder. UV–vis absorption diffuse reflectance spectra of H_2_SSeS, H_2_TzSeTz, UiO-68, **UiO-68-SSeS**, and **UiO-68-TzSeTz** in the solid state were recorded from 250 to 600 nm with a Jasco V-770 spectrophotometer equipped with a 60 mm integrating sphere and a PbS detector (ISN-923), using an interval wavelength of 1 nm. Fluorescence emission and excitation spectra of UiO-68, **UiO-68-SSeS**, and **UiO-68-TzSeTz** were recorded on sample powders at room temperature in a front-face acquisition geometry with a spectrofluorimeter (Fluorolog-3, Horiba Jobin Yvon) equipped with a double-grating monochromator in both the excitation and emission sides coupled to a R928P Hamamatsu photomultiplier and a 450 W Xe arc lamp as the excitation source. The emission spectra were corrected for detection and optical spectral response of the spectrofluorimeter as supplied by the manufacturer. The fluorescence excitation (FLE) spectra were corrected for the spectral distribution of the lamp intensity using a photodiode reference detector.

### 2.2 Synthesis of 2,5-Bis­(tributylstannyl)­selenophene (**4**—Scheme 2)

In a 50 mL oven-dried round-bottom flask under a nitrogen atmosphere, 2,5-dibromoselenophene (**3**) (0.40 mL, 1.00 g, 3.46 mmol, 1.0 equiv) was dissolved in anhydrous THF (16 mL), and the solution was cooled to 195 K. *n*-Butyllithium (1.6 M in hexanes, 5.2 mL, 8.3 mmol, 2.4 equiv) was added dropwise, and the reaction mixture was stirred at 195 K for 1.5 h. Tributyltin chloride (Bu_3_SnCl; 2.0 mL, 2.40 g, 7.38 mmol, 2.1 equiv) was then added dropwise, and the reaction mixture was allowed to warm slowly to room temperature under magnetic stirring overnight. Afterward, the reaction mixture was washed with water, followed by extraction with CH_2_Cl_2_. The combined organic layers were dried over anhydrous Na_2_SO_4_, filtered, and concentrated under reduced pressure to afford a yellowish oil (2.3 g), which was used in the subsequent step without further purification. ^1^H NMR (CDCl_3_, δ_Η_): 7.65 (s, 2H); 1.56 (m, 12H); 1.32 (m, 12H); 1.10 (m, 12H); 0.90 (m, 18H). ^13^C NMR (CDCl_3_, δ_C_): 148.6, 138.6, 29.0, 27.3, 13.6, 11.2. MS (*m*/*z*): 651 (M^•+^- Butyl).

**Scheme 2:**
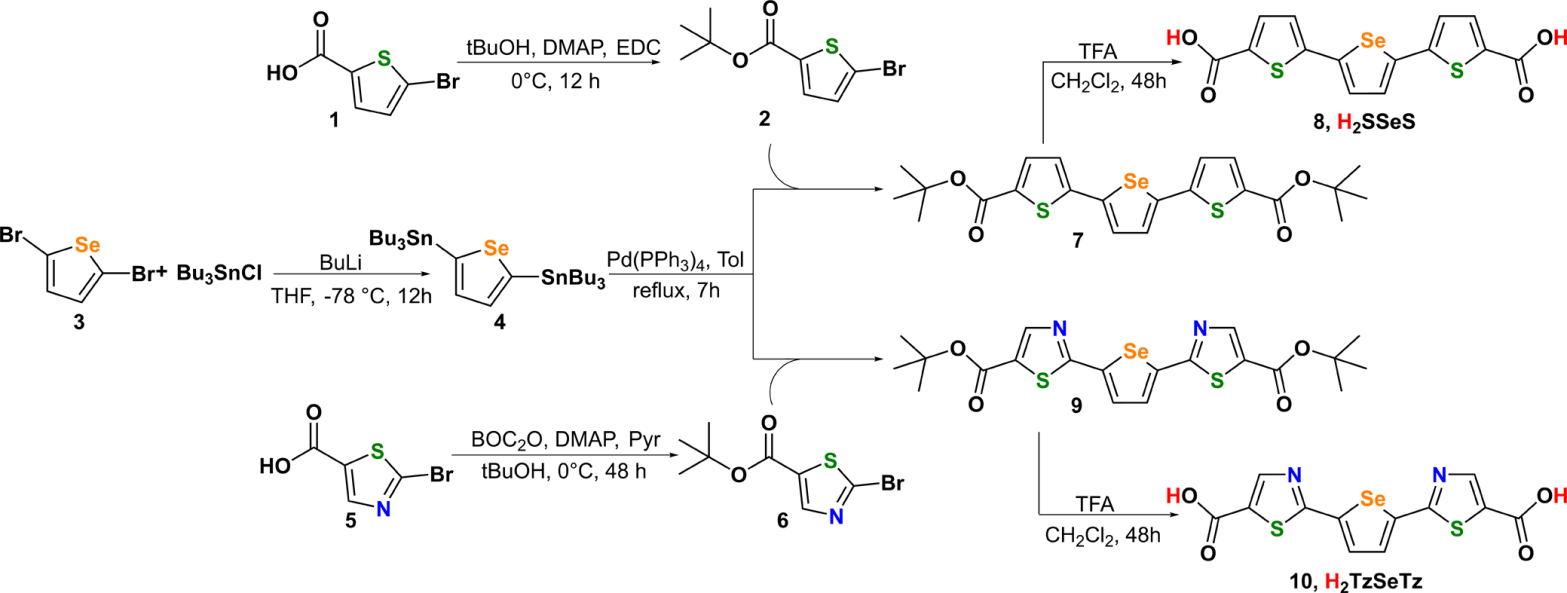
Overview of the Synthetic Path Leading to H_2_SSeS and H_2_TzSeTz

### 2.3 Synthesis of Di-*tert*-butyl 5,5′-(Selenophene-2,5-diyl)*bis*(thiophene-2-carboxylate) (**7**—Scheme 2)

*Tert*-butyl 5-bromothiophene-2-carboxylate (**2**) (1.00 g, 3.8 mmol, 2.1 equiv) was dissolved in anhydrous toluene (100 mL) in a 250 mL oven-dried round-bottom flask under a nitrogen atmosphere. Tetrakis­(triphenylphosphine)­palladium(0) [Pd­(PPh_3_)_4_] (175 mg, 0.15 mmol, 4 mol % with respect to the bromo derivative) was added, and the reaction mixture was heated to reflux. Subsequently, a solution of 2,5-bis­(tributylstannyl)­selenophene (**4**) (1.28 g, 1.8 mmol, 1.0 equiv) in anhydrous toluene (20 mL) was added dropwise. The reaction mixture was maintained at reflux for 7 h. Upon completion, a yellow solution was obtained. The solvent was removed under reduced pressure, and the crude residue was purified by column chromatography on silica gel, eluting with a gradient of pentane/ethyl acetate (from 95:5 to 90:10, v/v). The desired product was isolated as a yellow solid (655 mg, 73% yield). ^1^H NMR (CDCl_3_, δ_Η_): 7.60 (d, *J* = 4 Hz, 2H); 7.34 (s, 2H); 7.06 (d, *J* = 4 Hz, 2H), 1.50 (s, 18H). ^13^C NMR (CDCl_3_, δ_C_): 161.2, 144.8, 141.7, 134.1, 133.5, 127.9, 124.6 82.0, 28.2. MS (*m*/*z*): 496 (M^•+^).

### 2.4 Synthesis of 5,5′-(Selenophene-2,5-diyl)*bis*(thiophene-2-carboxylic acid) (H_2_SSeS, **8**—Scheme 2)

Di-*tert*-butyl 5,5′-(selenophene-2,5-diyl)­bis­(thiophene-2-carboxylate) (**7**) (406 mg, 0.819 mmol, 1.0 equiv) was dissolved in CH_2_Cl_2_ (8 mL), and trifluoroacetic acid (TFA, 0.6 mL, 9.5 equiv) was added. The reaction mixture was stirred at room temperature for 48 h, during which a yellowish precipitate formed. The precipitate was centrifugated, washed a few times with CH_2_Cl_2_, and then dried. The resulting solid was dissolved in 0.25 M aqueous NaOH (50 mL), and the diacid was reprecipitated by dropwise addition of concentrated HCl. After 3 h at 277 K, the supernatant was removed, and the solid was washed with deionized water until pH = 7 was reached. The product was oven-dried at 373 K and isolated as a yellow solid (283 mg, 90% yield). ^1^H NMR (DMSO-*d*_6_, δ_Η_): 7.64 (d, *J* = 4 Hz, 2H), 7.58 (s, 2H), 7.36 (d, *J* = 4 Hz, 2H).^13^C NMR (DMSO-*d*_6_, δ_C_): 163.0, 144.5, 141.4, 134.7, 133.9, 129.5, 126.3. MS (*m*/*z*): 384 (M^•+^). M.P. > 473 K. Single crystals of the hydrate sodium salt **Na**_2_ **SSeS·(H**_2_ **O)** suitable for X-ray diffraction analysis (Supporting Information and Figure S1) were obtained through slow evaporation of a basic (NaOH) aqueous solution of H_2_SSeS.

### 2.5 Synthesis of Di*-tert*-butyl 2,2′-(Selenophene-2,5-diyl)­bis­(thiazole-5-carboxylate) (**9**—Scheme 2)

*Tert*-butyl 5-bromothiazole-2-carboxylate (**6**) (0.56 g, 2.1 mmol, 2.5 equiv) was dissolved in anhydrous toluene (40 mL) in a 100 mL oven-dried round-bottom flask under a nitrogen atmosphere. Tetrakis­(triphenylphosphine)­palladium(0) [Pd­(PPh_3_)_4_] (146 mg, 0.126 mmol, 6 mol % with respect to the bromo derivative) was added, and the reaction mixture was heated to reflux. Subsequently, a solution of 2,5-bis­(tributylstannyl)­selenophene (**4**) (0.6 g, 0.85 mmol, 1.0 equiv) in anhydrous toluene (14 mL) was added dropwise. The reaction mixture was maintained at reflux for 7 h. Upon completion, an orange solution was obtained. The solvent was removed under reduced pressure, and the crude residue was purified by column chromatography on silica gel, eluting with pentane/ethyl acetate (95:5 v/v). The desired product was isolated as a yellow solid (161 mg, 38% yield). ^1^H NMR (CDCl_3_, δ_Η_): 8.23 (s, 2H), 7.72 (s, 2H), 1.59 (s, 18H). ^13^C NMR (CDCl_3_, δ_C_): 166.9, 160.5, 148.7, 145.2, 131.2, 130.3, 83.2, 28.4. MS (*m*/*z*): 498 (M^•+^).

### 2.6 Synthesis of 2,2′-(Selenophene-2,5-diyl)*bis*(thiazole-5-carboxylic acid) (H_2_TzSeTz, **10**—Scheme 2)

Di-*tert*-butyl 2,2′-(selenophene-2,5-diyl)­bis­(thiazole-5-carboxylate) (**9**) (269 mg, 0.54 mmol, 1.0 equiv) was dissolved in CH_2_Cl_2_ (13 mL), and trifluoroacetic acid (TFA, 0.8 mL, 25.4 equiv) was added. The reaction mixture was stirred at room temperature for 48 h, during which a yellowish precipitate formed. The precipitate was centrifugated, washed a few times with CH_2_Cl_2_, and then dried. The resulting solid was dissolved in 0.25 M aqueous NaOH (50 mL), and the diacid was reprecipitated by dropwise addition of concentrated HCl. After 3 h at 277 K, the supernatant was removed, and the solid was washed with deionized water until pH = 7 was reached. The product was oven-dried at 373 K and isolated as a yellow solid (169 mg, 81% yield). ^1^H NMR (DMSO-*d*_6_, δ_Η_): 8.31 (s, 2H), 8.06 (s, 2H). ^13^C NMR (DMSO-*d*_6_, δ_C_): 166.4, 162.3, 148.9, 144.3, 132.6, 131.6. MS (*m*/*z*): 386 (M^•+^). M.P.: 550 K, decomposition. Single crystals of the hydrate salt **(PPN)**_2_ **TzSeTz·6.6­(H**_2_ **O)** [PPN^+^ = bis­(triphenylphosphine)­iminium] suitable for X-ray diffraction analysis (Supporting Information and Figure S2) were grown at the interface of a layered water/methanol solution containing Na_2_TzSeTz and (PPN)­Cl, respectively. Na_2_TzSeTz was in turn prepared by dissolving H_2_TzSeTz in aqueous NaOH.

### 2.7 Synthesis of **UiO-68-SSeS**

Following the typical Solvent-Assisted Linker Exchange (SALE) procedure for the preparation of mixed-linker Zirconium MOFs,^29^ UiO-68 [with minimal formula Zr_6_O_4_(OH)_4_(TPDC)_4.6_(TFA)_2.8_, FW = 2450.9 g/mol, 0.100 g, 0.04 mmol] was suspended in a DMF solution (15 mL) of H_2_SSeS (FW = 383.31 g/mol, 0.046 g, 0.12 mmol, 3 equiv) and kept under gentle stirring at *T* = 393 K for 15 h. After that time, the suspension was cooled to room temperature, and the microcrystalline yellow powder of **UiO-68-SSeS·DMF** was collected, washed with EtOH (3 × 10 mL) and petroleum ether (3 × 10 mL), and finally dried under a nitrogen stream at room temperature. Yield: 0.080 g, 71%, based on the minimal formula [Zr_6_O_4_(OH)_4_(TPDC)_3.3_(TFA)_2.1_(SSeS)_1.65_]·3­(DMF) retrieved via thermogravimetric analysis (Section 3.2). Elemental analysis calcd. (%) for **UiO-68-SSeS**, C_93.3_H_53.5_O_32_F_6.3_S_3.3_Se_1.65_Zr_6_ (FW = 2589.66 g/mol): C, 43.3; H, 2.1; S, 4.1. Elemental analysis found (%): C, 43.0; H, 2.5; S, 4.0. IR bands (KBr pellet, cm^–1^): 1692­(sh) [ν­(COO)_TFA_], 1657(s) [ν­(COO)_SSeS_], 1602(s) [ν­(COO)_TPDC_], 1542­(m) [ν­(C=C)], 1409­(vs), 1186­(w), 1124­(w), 1004­(w), 830­(w), 768(s) [γ­(C–H)], 660­(w,br).

### 2.8 Synthesis of **UiO-68-TzSeTz**

UiO-68 [with minimal formula Zr_6_O_4_(OH)_4_(TPDC)_4.6_(TFA)_2.8_, FW = 2450.9 g/mol, 0.100 g, 0.04 mmol] was suspended in a DMF solution (15 mL) of H_2_TzSeTz (FW = 385.31 g/mol, 0.046 g, 0.12 mmol, 3 equiv) and kept under gentle stirring at *T* = 393 K for 15 h. After that time, the suspension was cooled to room temperature, and the microcrystalline yellow powder of **UiO-68-TzSeTz·DMF** was collected, washed with EtOH (3 × 10 mL) and petroleum ether (3 × 10 mL), and finally dried under a nitrogen stream at room temperature. Yield: 0.087 g, 80%, based on the minimal formula [Zr_6_O_4_(OH)_4_(TPDC)_2.4_(TFA)_2.4_(TzSeTz)_2.4_]·(DMF) retrieved via thermogravimetric analysis (Section 3.2). Elemental analysis calcd. (%) for **UiO-68-TzSeTz**, C_81.6_H_42.4_N_4.8_O_32_F_7.2_S_4.8_Se_2.4_Zr_6_ (FW = 2629.6 g/mol): C, 37.3; H, 1.6; N, 2.6; S, 5.8. Elemental analysis found (%): C, 37.5; H, 1.5; N, 2.9; S, 5.4. IR bands (KBr pellet, cm^–1^): 1698­(sh) [ν­(COO)_TFA_], 1635­(sh) [ν­(COO)_TzSeTz_], 1592(s) [ν­(COO)_TPDC_], 1552(s) [ν­(C=C)], 1406­(vs), 1288­(w), 1187­(w), 1147­(m), 1005­(w), 834­(w), 774(s) [γ­(C–H)], 643­(s,br).

### 2.9 Powder X-ray Diffraction Crystal Structure Characterization

To unveil the crystal structure of **UiO-68-SSeS** and **UiO-68-TzSeTz**, PXRD patterns were acquired in-house, under ambient conditions, in the 2θ range 1.5–105.0° and with steps of 0.013° (**UiO-68-TzSeTz**) or 0.02° (**UiO-68-SSeS**) using the diffractometer already quoted in Section 2.1. To the aim, a powdered sample of each MOF (*ca*. 20 mg) was deposited in the cavity of a silicon-free background sample holder. The subsequent data treatments were performed using the software TOPAS-R v.3.^30^ To preliminarily confirm the sample purity and crystallinity, a whole powder pattern refinement with the Le Bail method^31^ was performed. For **UiO-68-SSeS**, the cubic space group (*Fm*-3*m*) and unit cell parameter reported for UiO-68^32^ were employed. For **UiO-68-TzSeTz**, to describe all the observed Bragg reflections, a cubic space group of lower symmetry (*Pa*-3), together with a unit cell axis similar to that of pristine UiO-68, was adopted, as already done for a UiO-type MOF with the dibenzo­[*b*,*d*]­thiophene-3,7-dicarboxylato linker.^33^ In both cases, the background was described using a Chebyshev polynomial function, the instrumental contribution to the peak profile was modeled using the fundamental parameters approach,^34^ while the sample contribution to the peak profile was described using a convolution of Lorentzian and Gaussian functions. Spherical harmonics were employed to model anisotropic peak broadening. The crystallographically independent portion of the linkers was built as a rigid group using the z-matrix formalism adopting idealized bond distances and angles.^35^ The position and orientation of the rigid groups were set according to the reported UiO-68 crystal structure, assigning to their centers of mass the [0.25, 0, 0.25] fractional coordinates (for **UiO-68-SSeS**, space group *Fm*-3*m*, they correspond to a special position with Wyckoff letter *d*). The crystallographic symmetry is such that the two rigid bodies describing TPDC^2–^ and the heteroaromatic linker are superimposed, this occurence resulting in a uniform distribution of the heterocyclic and carbocyclic linkers inside the crystal structure. The site occupation factor (s.o.f.) of the vicariant TPDC^2–^ and heteroaromatic linker was initially assigned based on the molar ratio retrieved by ^1^H NMR spectroscopy of the digested MOF. For **UiO-68-SSeS**, based on the symmetry operations of space group *Fm*-3*m* (implying 1/4 of the linker as crystallographically independent portion) and the molecular structure of SSeS^2–^, half of this linker was built as a rigid group, thus implying a random orientation of the pentatomic rings inside the crystal structure, and its site occupation factor was halved. The fractional coordinates of the crystallographically independent zirconium and oxygen atoms forming the inorganic nodes were initially assigned based on the reported UiO-68 crystal structure and were then refined. For simplicity, both the μ^3^-O^2–^ and μ^3^-OH^–^ ligands were described only as oxygen atoms. A single isotropic thermal parameter *B*_iso_ was assigned to the Zr^IV^ ions, while the isotropic thermal parameter of all the other atoms was calculated as *B*_iso_(Zr^IV^) + 2 Å^2^. To obtain a better agreement between the observed and calculated PXRD patterns, for both MOFs, the presence of missing linker defects was supposed. To verify or confute this hypothesis, the two linkers s.o.f.s were refined independently, without correlating them based on the ratio retrieved through ^1^H NMR spectroscopy, which was employed only to limit their maximum values. The results confirmed the 1:1 linker ratio retrieved via ^1^H NMR for **UiO-68-TzSeTz**, with a percentage of missing linkers of about 20%. At variance, for **UiO-68-SSeS**, a TPDC^2–^:TzSeTz^2–^ ratio amounting to 4:1.1 was retrieved which, together with the 15% of missing linkers, was maintained in the subsequent steps. The missing charge was counterbalanced using TFA anions, deriving from the usage of TFA as a modulator in the synthesis of the parent UiO-68 MOF. TFA presence was confirmed by ^19^F NMR spectroscopy. As two rigid bodies were already superimposed in the crystal structures, for simplicity, the TFA molecule was built binding the fluorine atoms to the carbon atom of the TPDC^2–^ linker bound to the carbon atom of the carboxylate group. The site occupation factors of the vicariant atoms were then adjusted to ensure an overall charge balance in the resulting minimal formula. The final stage of the structure refinement was carried out using the Rietveld method.^36^ At this stage, all the variables describing the instrumental and sample contributions to the peak profile, the unit cell axis, and the coefficients of the polynomial function describing the background were collectively refined. In **UiO-68-TzSeTz**, both the phenyl rings of TPDC^2–^ and the heterocyclic rings of TzSeTz^2–^ were allowed to rotate around their axes. In **UiO-68-SSeS**, only the phenyl rings of TPDC^2–^ were allowed to rotate around the molecular axis, thus introducing rotational disorder; the rotation of the pentatomic rings around the molecular axis was not allowed as it caused a nonrealistic distortion of the linker bond angles.

Crystallographic information for **UiO-68-SSeS**, Zr_6_O_4_(OH)_4_(TPDC)_4_(SSeS)_1.1_(TFA)_1.8_, C_99_H_58.6_F_5.4_O_32_S_2.2_Se_1.1_Zr_6_, FW = 2567.48 g mol^–1^, cubic, *Fm*-3*m*, *a* = 32.864(1) Å, *V* = 35,496(3) Å^3^, *Z* = 4, *Z*′ = 0.02, ρ = 0.48 g cm^–3^, *F*(000) = 5079, *R*_Bragg_ = 1.2%, *R*_p_ = 3.2%, *R*_wp_ = 4.1%, for 5087 data and 51 parameters in the 3.25°–105.0° 2θ range. Cambridge Crystallographic Data Centre (CCDC) number: 2532999.

Crystallographic information for **UiO-68-TzSeTz**, Zr_6_O_4_(OH)_4_(TPDC)_2.4_(TzSeTz)_2.4_(TFA)_2.4_, C_81.6_H_42.4_N_4.8_O_32_F_7.2_S_4.8_Se_2.4_Zr_6_, FW = 2629.6 g/mol, cubic, *Pa*-3, *a* = 32.896(1) Å, *V* = 35,598(5) Å^3^, *Z* = 4, *Z*′ = 0.17, ρ = 0.49 g cm^–3^, *F*(000) = 5139, *R*_Bragg_ = 0.5%, *R*_p_ = 2.1%, *R*_wp_ = 2.7%, for 7846 data and 55 parameters in the 3.0°–105.0° 2θ range. CCDC number: 2533000.

### 2.10 Textural Properties Assessment through N_2_ Adsorption

Powdered samples (ca. 40 mg) of UiO-68, **UiO-68-SSeS**, and **UiO-68-TzSeTz** were activated at *T* = 403 K under high vacuum (10^–6^ Torr) for 24 h before the measurement. The Brunauer–Emmett–Teller (BET) specific surface area (SSA), pore size distribution, and pore volume (*V*_tot_, V_micro_) were estimated by volumetric adsorption with an ASAP 2020 Micromeritics instrument, using N_2_ as the adsorbate at *T* = 77 K. For the BET SSA calculation, the 0.01–0.1 *p*/*p*_0_ pressure range of the isotherm was used to fit the data. Within this range, all the Rouquerol consistency criteria^37^ are satisfied. The material (micro)­porosity was determined from the N_2_ adsorption isotherm using a NLDFT method (Tarazona approximation) and assuming a cylindrical pore shape (typical of metal oxides).

### 2.11 Photophysical Characterization by Means of Confocal Fluorescence Microscopy

Fluorescence confocal imaging was performed on an inverted Nikon Ti-E microscope (Nikon Co., Tokyo, Japan). The confocal fluorescence microscope Nikon A1 is equipped with an argon ion CW laser as well as 405 and 485 nm pulsed diode lasers (PicoQuant GmbH, Berlin, Germany). Images were collected using a Nikon Plan Apo VC 60× objective with an NA of 0.9. Filters were set to register fluorescence in the 500–540, 560–630, and 660–740 nm ranges. Spectral imaging was performed with the Nikon spectral module of 32 PMT setting a range of 10 nm per detector; the samples were excited at either 488 or 405 nm. Fluorescence lifetime imaging was performed by exciting with a pulsed 405 or 485 nm diode laser and collecting photons with integrated PicoHarp 300 electronics (PicoQuant GmbH, Berlin, Germany) for TCSPC measurements. A single-photon avalanche diode detector equipped with a bandpass filter was used as a detector. Bandpass filters centered at 480 and 520 nm were used for 405 nm excitation and at 520 and 585 nm for 485 nm excitation. Histograms of collected photons consist of 3200 channels, each with 16 ps width. The repetition rate of pulsed excitation was 40 MHz. The instrument response function of the system was approximately 220 ps. The fluorescence decay fit was performed on the histogram calculated for a region of interest. The fluorescence decay profile was analyzed with a least-squares method using a biexponential decay function provided by the Picoquant SymPhoTime software.

The fitting function used is

1 $$I\left(t\right)=b+\Sigma_{j}a_{j}\exp\left({-t/\tau }_{j}\right)$$

The fractional intensity (*f*_i_) and the average fluorescence lifetime (τ_av_) are calculated according to the following equations:

2 $$f_{i}=a_{i}\tau_{i}/\Sigma_{j}a_{j}\tau_{j}$$

3 $$\tau_{av}=\Sigma_{j}f_{j}\tau_{j}$$

### 2.12 PFAS Adsorption and Quantification

5 mg of MOF (UiO-68, **UiO-68-SSeS**, or **UiO-68-TzSeTz**) was sonicated in 9.95 mL of tap water (Bologna municipality, Italy) for 2 h. A mixture containing six PFAS (50 μL, 100 μg/L) was then added to the suspensions to achieve a final concentration of 0.5 μg/L each PFAS in a total volume of 10 mL. All the samples were subjected to gentle agitation for 24 h, then centrifuged (10,000 rpm, 10 min), and filtered on 0.2 RC Sartorius filters. The residual concentration of PFAS was determined by UPLC-MS/MS analysis (ACQUITY UPLC H-Class PLUS coupled to a XEVO TQS Micro mass detector, Waters). A 1 mL aliquot of each sample was used for automated injection. Chromatographic separation was performed on a Waters Acquity UPLC CSH Phenyl-Hexyl column (1.7 μm, 2.1 × 100 mm) with a Waters Isolator Column (2.1 × 50 mm). The column temperature was maintained at 307 K, the flow rate was 0.3 mL min^–1^, and the injection volume was 40 μL. Total run times were 8 min for perfluorobutanoic acid (PFBA) and 21 min for the mixture of six PFAS. The mobile phase consisted of a biphasic gradient: 2 mM ammonium acetate (NH_4_OAc) in ultrapure water/methanol (95:5) as phase A and 2 mM NH_4_OAc in methanol as phase B (for further details, see the Supporting Information). The experiments were repeated in triplicate, and results are reported as mean values.

## 3 Results and Discussion

### 3.1 Synthesis and Characterization of H_2_SSeS and H_2_TzSeTz

The synthesis of the selenophene-based diacid trimers H_2_SSeS and H_2_TzSeTz was achieved through a Stille cross-coupling reaction involving 2,5-bis­(tributylstannyl)­selenophene (**4**) and the corresponding brominated heterocycles, namely, 2-bromo-5-*tert*-butyl thiophene-2-carboxylate (**2**) or 2-bromo-5-*tert*-butyl thiazole-2-carboxylate (**6**). The coupling reactions were performed in refluxing toluene using tetrakis­(triphenylphosphine)­palladium(0) [Pd­(PPh_3_)_4_] as the catalyst. Compounds **2** and **6** were synthesized according to previously reported procedures and obtained in 61% and 86% yields, respectively.^38^ The key intermediate 2,5-bis­(tributylstannyl)­selenophene (**4**) was prepared from commercially available 2,5-dibromoselenophene via lithiation followed by stannylation with Bu_3_SnCl in THF and used without purification in the subsequent step. Stille cross-coupling of compound **4** with brominated derivatives **2** and **6** afforded the corresponding diester trimers **7** and **9** in 73% and 38% yields, respectively. Final conversion to the diacid trimers, H_2_SSeS (**8**) and H_2_TzSeTz (**10**), was accomplished by *tert*-butyl ester deprotection of intermediates **7** and **9** using TFA in CH_2_Cl_2_, providing the diacids in 90% and 81% yields, respectively.

### 3.2 Synthesis and Characterization of **UiO-68-SSeS** and **UiO-68-TzSeTz**

The mixed-linker Zirconium MOFs **UiO-68-SSeS** and **UiO-68-TzSeTz** were prepared through the classical SALE procedure commonly exploited with this class of materials.^39–41^ The starting MOF UiO-68, bearing the fully carbocyclic TPDC^2–^ linker and prepared using TFA as a crystal modulator, shows a defective nature, with some TFA anions replacing TPDC^2–^ on the metal nodes. Incidentally, the formation of missing-linker defects in UiO-type MOFs synthesized using TFA as a modulator was already observed for UiO-66 in the past.^42^ In our UiO-68, the presence of the trifluoroacetate linker is confirmed by its typical ^19^F NMR resonance falling at δ_F_ ≈ −75 ppm and found in the spectrum of a digested sample (Figures S3 and S4). An accurate defects quantification in the homemade UiO-68 was performed through (preactivated) sample digestion in an acidic D_2_SO_4_/D_2_O solution followed by a combined ^1^H/^19^F NMR spectroscopy analysis, using 2,6-difluorobenzoic acid as an internal standard. Based on these results, we propose the minimal formula [Zr_6_O_4_(OH)_4_(TPDC)_4.6_(TFA)_2.8_] for the batch of UiO-68 used in this work. UiO-68 was suspended in a DMF solution containing either H_2_SSeS or H_2_TzSeTz and kept in this suspension under stirring overnight at *T* = 393 K, slightly below its synthesis temperature (403 K). During this time, part of TPDC^2–^ and/or the TFA anion were replaced by the heterocyclic spacers, forming the corresponding MIXMOF in reasonably high yields (71–80%) and keeping the same **fcu** cubic topology of all the members of the UiO-6x family. Remarkably, direct solvothermal synthesis starting from either Zirconium chloride (ZrCl_4_) or oxychloride (ZrOCl_2_) in DMF in the presence of both linkers in a 6:3:3 relative stoichiometric ratio was unsuitable for the preparation of a pure and microcrystalline phase, as judged by PXRD qualitative analysis of the recovered solids. IR spectroscopy (Figures S5 and S6) confirms the presence of both linkers in the material besides TFA, considering the typical [ν­(COO)]_*asym*_ vibrational modes of TPDC^2–^ (1592/1602 cm^–1^), SSeS^2–^/TzSeTz^2–^ (1657/1635 cm^–1^), and TFA (1692/1698 cm^–1^). The presence of TFA was also proven through ^19^F NMR spectroscopy of the digested samples (Figure S7). The relative amount of the two aromatic linkers in the samples was assessed through digestion of the preactivated solid in D_2_SO_4_/D_2_O/DMSO-*d*_6_ at *T* = 353 K for 2 h and followed by ^1^H NMR spectroscopy data collection on the clear solution. ^1^H NMR signal integration of the two linkers resonances led to a 2:1 and 1:1 relative carbocyclic:heterocyclic linker stoichiometric ratio for **UiO-68-SSeS** and **UiO-68-TzSeTz**, respectively (Figures S8 and S9). Thermogravimetric analysis (Figures S10 and S11) unveiled the high thermal stability of the MIXMOFs, with a decomposition temperature *T*_dec_ = 818 and 804 K (for **UiO-68-SSeS** and **UiO-68-TzSeTz**, respectively), slightly lower than that of UiO-68 (*T*_dec_ = 833 K, Figure S12).^23^ In **UiO-68-SSeS**, a first weight loss of ca. 7.0 wt % centered at *T* ∼ 490 K can be ascribed to DMF {theoretical weight loss for [Zr_6_O_4_(OH)_4_(TPDC)_3.3_(TFA)_2.1_(SSeS)_1.65_]·3­(DMF) = 7.8 wt %}, in agreement with the presence of its mass peak at *m*/*z* = 73 a.m.u. in the mass spectrum of the volatiles. A second thermal event centered at *T* ∼ 690 K (weight loss of ca. 28.0 wt %) can be reasonably ascribed to the SSeS^2–^ linker decomposition (theoretical weight loss = 22.4 wt %). From an independent TG-DTG measurement, it was found that H_2_SSeS shows a decomposition peak at *T* = 600 K in the DTG profile. Further proof of evidence comes from the MS spectrum of the volatiles, where a peak at *m*/*z* = 84 a.m.u. (thiophene) appears in the same temperature range. After the degradation of the heterocyclic linker, the MOF decomposes at the above quoted *T*_dec_; a peak at *m*/*z* = 78 a.m.u. (benzene) on the MS spectrum confirms the degradation of the TPDC^2–^ linker at the last thermal event. A similar thermal behavior is observed for **UiO-68-TzSeTz** as well: after an initial weight loss of ca. 3.0 wt % centered at *T* = 490 K and ascribed to DMF {theoretical weight loss for [Zr_6_O_4_(OH)_4_(TPDC)_2.4_(TFA)_2.4_(TzSeTz)_2.4_]·(DMF) = 2.7 wt %; *m*/*z* = 73 a.m.u. in the mass spectrum of the volatiles}, the heterocyclic linker decomposition occurs at *T* = 670 K (experimental weight loss = 30.0 wt %; theoretical weight loss = 34.0 wt %, *m*/*z* = 85 a.m.u. of thiazole in the mass spectrum of the volatiles. Free H_2_TzSeTz decomposes at *T* = 550 K, as assessed through a dedicated TG-DTG analysis). MOF degradation occurs at the above quoted *T*_dec_ (*m*/*z* = 78 a.m.u. of benzene in the mass spectrum of the volatiles). The presence of TFA on the defects is witnessed in both cases by the presence of an additional mass peak at *m*/*z* = 45 a.m.u. coming from trifluoroacetic acid fragmentation at the main thermal events. The comprehensive information coming from the aforementioned characterization techniques (also combined with that coming from the elemental analysis) led to the minimal formulas [Zr_6_O_4_(OH)_4_(TPDC)_3.3_(TFA)_2.1_(SSeS)_1.65_]·3­(DMF) and [Zr_6_O_4_(OH)_4_(TPDC)_2.4_(TFA)_2.4_(TzSeTz)_2.4_]·(DMF) for the as-synthesized **UiO-68-SSeS**·**DMF** and **UiO-68-TzSeTz**·**DMF**, respectively. These formulas are representative of the whole sample (which comprises the microcrystalline MOF phase and a minor fraction of amorphous phase; see Section 3.3 and Figure S13). Remarkably, the presence of residual TFA anions anchored at the nodes in both MOFs should not be considered as a detrimental factor for the applicative context under investigation, as it has already been demonstrated that the functionalization of MOF-808 with TFA improves its PFAS adsorption performance.^43^

### 3.3 Structural Features of **UiO-68-SSeS** and **UiO-68-TzSeTz**

The crystal structure of **UiO-68-SSeS** and **UiO-68-TzSeTz** was disclosed by in-house PXRD. Figure S13 proposes the final stage of the structure refinements carried out with the Rietveld method, while Figures 1 and S14 show the crystal structure of **UiO-68-TzSeTz** and **UiO-68-SSeS**, respectively. As proved by a preliminary whole powder pattern refinement followed by the successful structure characterization, **UiO-68-SSeS** shares the same cubic space group (*Fm*-3*m*) of pristine UiO-68, while **UiO-68-TzSeTz** crystallizes in a cubic space group of lower symmetry, *Pa*-3. The symmetry decrease with respect to the parent material was already observed in UiO-6x-type MOFs containing linkers featuring pentatomic rings, for example, UiO-66-PZDC^44^ and UiO-67 S.^33^ Both MOFs possess a unit cell axis length [*a* = 32.864(1) and 32.896(1) Å for **UiO-68-SSeS** and **UiO-68-TzSeTz**, respectively] comparable to that of pristine UiO-68 [*a* = 32.777(<1) Å at *T* = 173 K].^32^ The symmetry imparted by the space groups employed for the structural characterization generates a uniform distribution of the two vicariant linkers (and TFA anions counterbalancing the missing linkers). The overall crystal structure of the two MIXMOFs (Figures 1a and S14a) resembles that of pristine UiO-68 and features 12-connected octahedral [Zr_6_O_4_(OH)_4_]^12+^ nodes (Figures 1b and S14b) on average bridged by ditopic TPDC^2–^, SSeS^2–^, or TzSeTz^2–^ linkers, resulting in a framework of **fcu** topology characterized by tetrahedral and octahedral cavities (Figures 1c,d and S14c,d) with a diameter of ca. 8 and 16 Å, respectively.^45^ As explained in Section 2.9, we verified the persistence of missing-linker defects, already witnessed in pristine UiO-68. At this stage, the site occupation factors of the carbocyclic and heterocyclic linkers were refined without correlating them based on the ratio retrieved by ^1^H NMR spectroscopy. Performing this refinement, we disclosed that, while for **UiO-68-TzSeTz** the linker ratio 1:1 was maintained, for **UiO-68-SSeS**, a different TPDC^2–^: SSeS^2–^ratio (4.0:1.1) was obtained, confirming the hypothesis that part of the SSeS^2–^ spacer is contained in the amorphous fraction of the sample. In addition, both MOFs are characterized by missing linker defects amounting to 15 and 20% for **UiO-68-SSeS** and **UiO-68-TzSeTz**, respectively, leading to the minimal formulas [Zr_6_O_4_(OH)_4_(TPDC)_4_(SSeS)_1.1_(TFA)_1.8_] and [Zr_6_O_4_(OH)_4_(TPDC)_2.4_(TzSeTz)_2.4_(TFA)_2.4_], respectively, exclusively accounting for the microcrystalline fraction. Incidentally, this outcome suggests that in both MOFs, the heterocyclic linker partially replaces not only TPDC^2–^ but also the TFA anions, albeit with different extents. In the case of **UiO-68-SSeS**, a higher missing-linker defect remediation is observed. Disregarding the presence of missing linkers, the empty volume equals 67% and 70% for **UiO-68-SSeS** and **UiO-68-TzSeTz**, respectively.^46^ These values are lower than that retrieved for the crystal structure of UiO-68 published by Manna et al.^28^ (76.8% at *T* = 100 K): the discrepancy could be reasonably attributed to the rotational disorder affecting the linkers in the MIXMOFs, causing the carbocyclic and heterocyclic rings to protrude inside the cavities. Indeed, a lower empty volume (74.7% at *T* = 173 K) was estimated for UiO-68 as reported by Schaate et al.,^32^ in which the central phenyl ring of TPDC^2–^ was affected by rotational disorder. In **UiO-68-SSeS** and **UiO-68-TzSeTz**, both the central and lateral phenyl rings of TPDC^2–^ are rotated with respect to the plane where the atoms of the carboxylate groups lay (Table S3). In **UiO-68-TzSeTz**, the central and lateral pentatomic rings of TzSeTz^2–^ are rotated as well (Table S3), while no rotational disorder was introduced for the heterocyclic linker in **UiO-68-SSeS**. As expected, the estimated empty volumes for the ideal crystal structures of **UiO-68-SSeS** and **UiO-68-TzSeTz** after removal of the rotational disorder are higher (77% and 78%, respectively).

**Figure 1:**
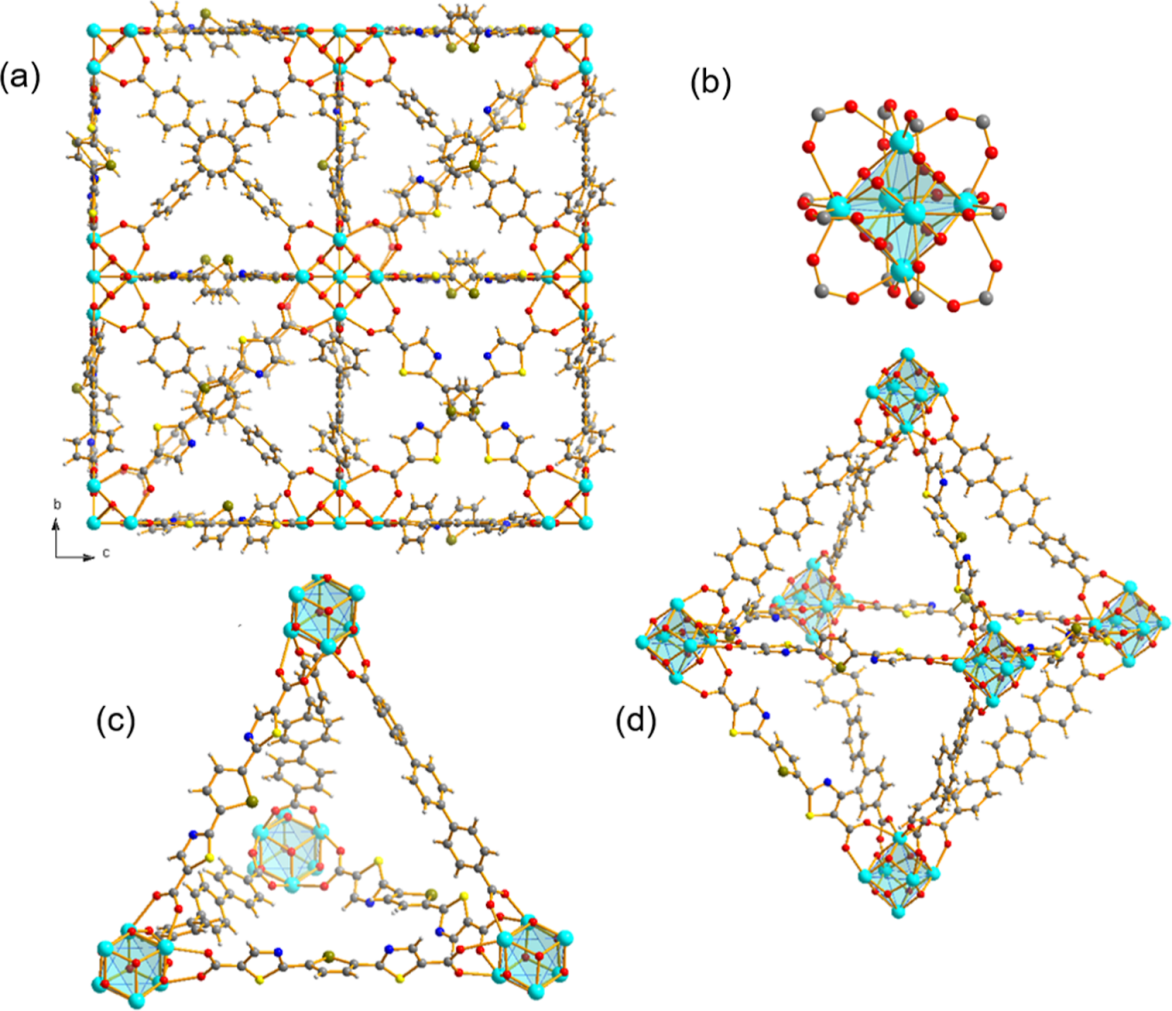
Representation of the crystal structure of **UiO-68-TzSeTz**: (a) portion of the crystal structure viewed along one of the unit cell axes; (b) the node; (c) the tetrahedral cavity; and (d) the octahedral cavity. For simplicity, an ideal formula disregarding missing linker defects was adopted. Element color code: C, dark gray, H, light gray; N, blue; O, red; S, yellow; Se, gold; Zr, cyan. For the representation of the crystal structure of **UiO-68-SSeS**, the reader is addressed to Figure S14.

### 3.4 Textural Properties Assessment

The porosity of **UiO-68-SSeS** and **UiO-68-TzSeTz** was analyzed through N_2_ volumetric adsorption at *T* = 77 K on preactivated powdered samples (Figure 2). The isotherm shapes are of Type I, typical of microporous materials. The BET SSAs equal 1242 and 550 m^2^/g for **UiO-68-SSeS** and **UiO-68-TzSeTz**, respectively. They are much lower than those of UiO-68 prepared with the “Lin synthetic protocol” followed by us (2815 m^2^/g)^28^ or Zr_BTDZ (3770 m^2^/g), a parent mixed-linker compound derived from UiO-68 and made of TPDC^2–^ and BTDZ^2–^ (H_2_BTDZ = 4,4′-(benzothiadiazole-4,7-diyl)­dibenzoic acid).^25^ If we take the BET SSA of our “home-made” UiO-68 as a reference (950 m^2^/g, Figure S15), we observe an opposite trend for the two MIXMOFs. While for **UiO-68-SSeS**, a surface area increase is observed after SALE; in **UiO-68-TzSeTz**, a decrease is instead recorded. This confirms that SSeS^2–^ replaces TFA “repairing” the pristine UiO-68 defects and thus increasing the initial SSA, while TzSeTz^2–^ preferentially replaces TPDC^2–^, causing a SSA reduction with respect to UiO-68. The low value found for **UiO-68-TzSeTz** may also stem from its lower thermal stability with respect to **UiO-68-SSeS** (Section 3.2) and the higher amount of amorphous phase in its sample (Figure S13b). The micropore size distribution evaluated through NLDFT methods has disclosed the presence of micropores of ca. 18, 22, and 26 Å size in both MIXMOFs (Figure S16). The additional pore size with respect to the bimodal distribution normally expected for a nondefective UiO-68 (deriving from the two types of cavities of the 3D framework) is reasonably due to the presence of missing linkers (§3.3).

**Figure 2:**
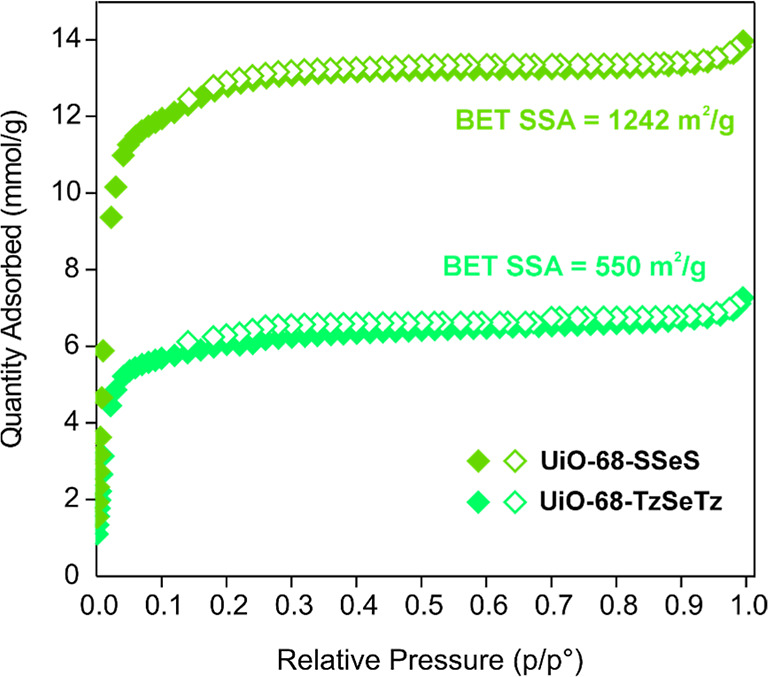
N_2_ adsorption isotherms measured at 77 K of **UiO-68-SSeS** and **UiO-68-TzSeTz** at comparison. Empty symbols denote desorption branches.

### 3.5 Luminescence Properties of **UiO-68-SSeS** and **UiO-68-TzSeTz**

The luminescence properties of **UiO-68-SSeS** and **UiO-68-TzSeTz** were studied in the solid state (Figure 3) and compared with those of the parent MOF UiO-68. Under UV excitation, UiO-68 exhibits intense blue-violet fluorescence centered at λ = 420 nm and extending up to 600 nm. The corresponding excitation spectrum is broad and primarily confined to the UV region, with a maximum at λ = 370 nm and a low-intensity tail extending into the visible range.

**Figure 3:**
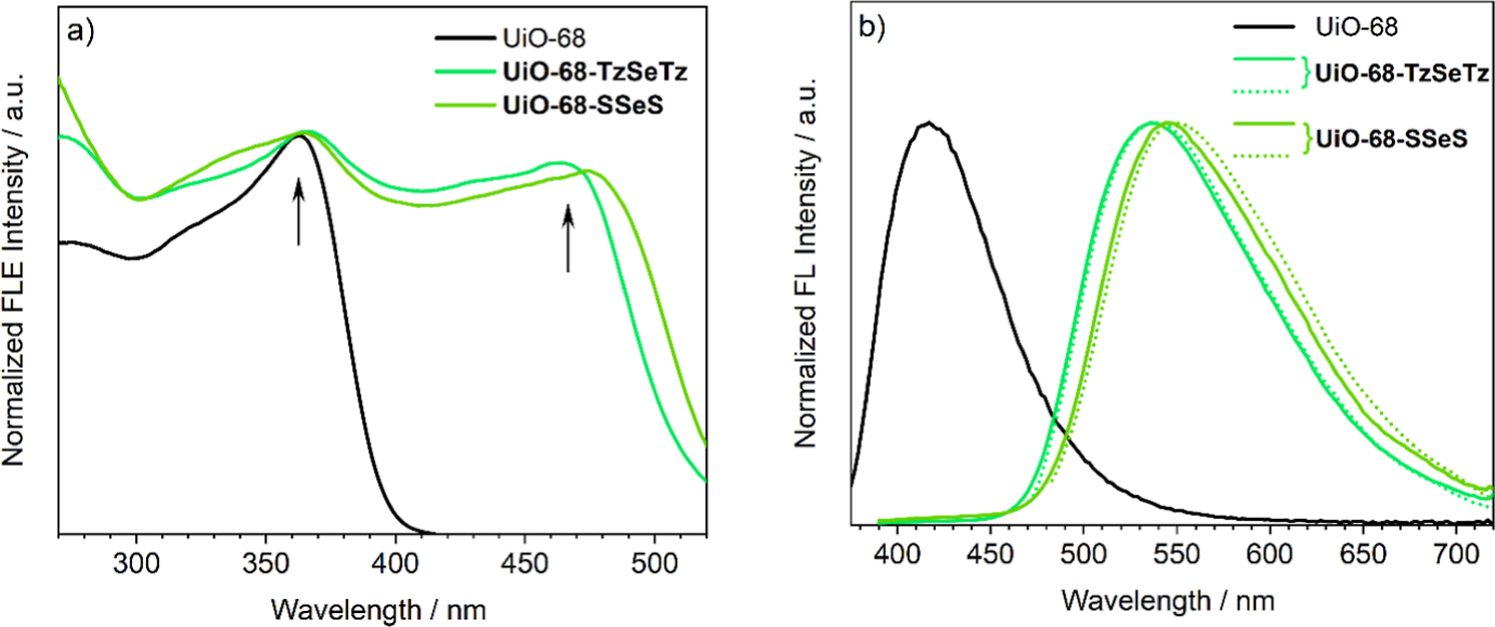
(a) FLE spectra of powdered samples of UiO-68, **UiO-68-SSeS**, and **UiO-68-TzSeTz** monitored at the maximum emission wavelength. Arrows indicate the excitation wavelengths for the fluorescence spectra shown in panel b. (b) Fluorescence spectra of MOF powders excited at λ_exc_ = 370 nm (solid line) and 465 nm (dotted line).

UiO-68 modification through the introduction of the selenophene-based linkers significantly alters both the excitation and emission profiles. Specifically, the fluorescence spectra exhibit a red-shift of more than 100 nm, while the excitation spectra extend well into the visible region beyond 500 nm, with a maximum at λ = 465 nm. The fluorescence bands for **UiO-68-SSeS** and **UiO-68-TzSeTz** are centered at λ = 546 nm and λ = 538 nm, respectively, with an olive green or bright green emission color, respectively. The corresponding CIE diagrams are reported in Figure 4. No appreciable changes in the emission profile are observed when scanning the excitation wavelength across the entire range. Furthermore, upon excitation at 370 nm, a common maximum for all the investigated materials, no residual emission from the parent UiO-68 MOF is detected.

**Figure 4:**
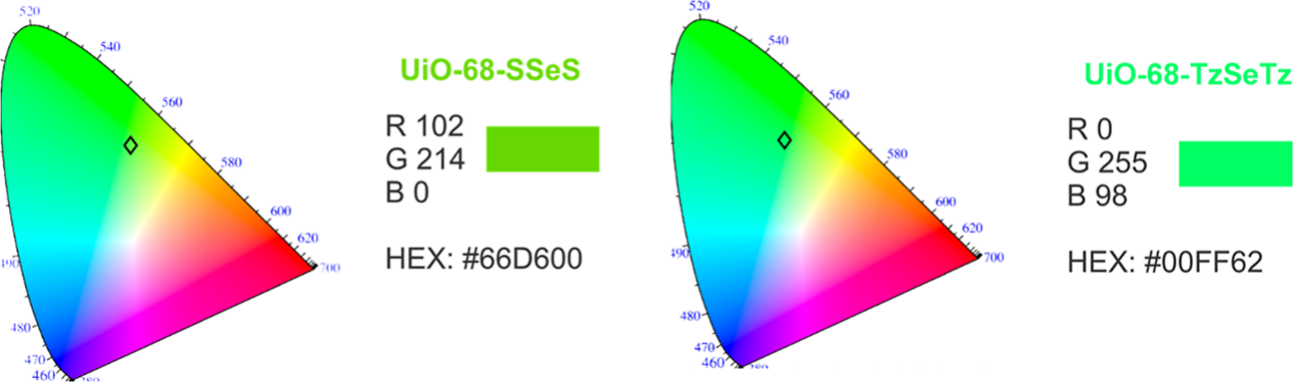
CIE diagrams of **UiO-68-SSeS** and **UiO-68-TzSeTz** derived from the emission spectra in Figure 3, with hexadecimal (HEX) and RGB coordinates of the corresponding emission color.

The MOFs have been further characterized through confocal fluorescence microscopy including spectral and lifetime imaging. For excitation at λ = 405 nm, the lowest accessible wavelength available, UiO-68 is characterized by a spectrum that extends from λ = 425 nm to λ = 600 nm with maximum at 440 nm (Figure S17). Fitting of the fluorescence decay at λ = 480 or λ = 520 nm of a selected area requires a biexponential function affording two lifetimes of 0.8 and 3.8 ns (Figure S18). The mean lifetime is 2.5 ns for the fitted decay. The fluorescence lifetime is very sensitive to the local environment that may be influencing decay dynamics giving origin to multiexponential decays. The differences in local environment may also explain why the confocal spectrum does not fully correspond to the bulk emission spectrum obtained by exciting at 370 nm, wavelength not accessible in the confocal setting. As for the MIXMOFs, the spectral images evidence different *loci* in the material where the emitting units likely experience different microenvironments that can give origin to fluorescence spectra with changing features. Indeed, two emissive units compose the material, the heterocyclic and the carbocyclic aromatic linker. These features may not emerge in bulk measurements where only a single emission peak for excitation at λ = 370 nm is dominating (Figure 3), losing the spatial information. As for **UiO-68-SSeS** (Figure 5), spectral imaging upon excitation at λ = 405 nm clearly reveals two types of fluorescence: one band peaking at λ = 530 nm ascribable to the SSeS^2–^ heterocyclic linker [whose solid-state emission is centered at λ = 526 nm (Figure S19)] and the other blue-shifted band with maximum at λ ≈ 440 nm likely due to TPDC^2–^ emission also present in UiO-68. For excitation at λ = 488 nm, we still observe the band peaking at λ = 530 nm. The average fluorescence lifetime is very short: 0.22 and 0.30 ns at λ = 520 nm and λ = 580 nm, respectively. In **UiO-68-TzSeTz** (Figure S20), excitation at λ = 405 and λ = 488 nm provides the same fluorescence features. We observe two main emissions, peaking at λ = 490 nm (TPDC^2–^) and λ = 520 nm (TzSeTz^2–^, λ_em_ = 556 nm, Figure S19). For excitation at λ = 405 nm, two short lifetimes of 0.2 and 0.8 ns are obtained with an average lifetime of 0.25 ns. In all cases, the simultaneous presence of carbocyclic and heterocyclic linkers in the solid phase gives rise to two main emission bands and multiexponential decays. The short lifetimes are in line with other lifetime data for thiophene-based materials.^47,48^

**Figure 5:**
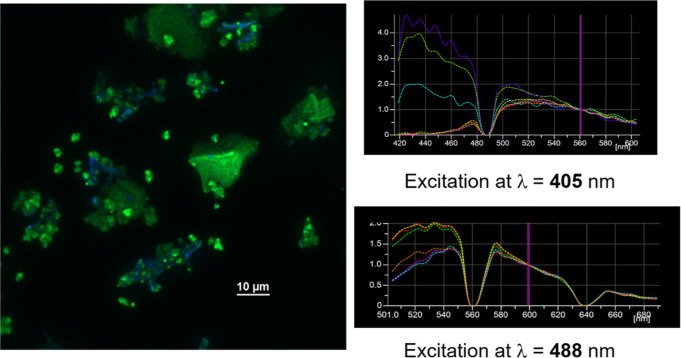
**UiO-68-SSeS** confocal image and spectra.

### 3.6 PFAS Adsorption Tests in Tap Water

**UiO-68-TzSeTz** and **UiO-68-SSeS** were evaluated as sorbents toward tap water samples spiked with a mixture of six PFAS, differing in fluorinated chain length [(CF)_6–10_] and terminal functional groups (sulfonates and carboxylates). The adsorption performance was compared under the same experimental conditions to that of UiO-68, parent material taken as a reference. The PFAS concentrations were selected to reflect environmentally relevant levels commonly detected in contaminated water (0.1–3 μg/L),^49,50^ and the experiments were conducted at the native pH of tap water (pH ≈ 7). Both MOFs are chemically stable in water, even if a prolonged exposure to this solvent causes a partial crystallinity loss, as evidenced by their PXRD patterns collected before and after adsorption at comparison (Figures S21 and S22). The adsorption contact time was fixed at 24 h, and the removal efficiency of each PFAS was quantified through UPLC-MS/MS analysis (see the Supporting Information). The removal efficiency after 24 h is shown in Figure 6 and reveals a clear dependence from the pollutant chain length for both **UiO-68-TzSeTz** and **UiO-68-SSeS**. Shorter-chain PFAS [namely, perfluorohexanesulfonic acid (PFHxS) and perfluorooctanoic acid (PFOA)] were poorly adsorbed, with removal efficiencies in the range of 8–12% for both materials. A marked increase in adsorption performance was observed starting from perfluorononanoic acid (PFNA), which is removed with efficiencies of 24% and 29% by **UiO-68-TzSeTz** and **UiO-68-SSeS**, respectively. Further performance enhancement is evident for longer-chain PFAS [(CF)_8–10_]; indeed, perfluorooctanesulfonic acid (PFOS) and perfluorodecanoic acid (PFDA) are comparably removed by both MOFs, displaying efficiencies between 43% and 58%. Notably, in the case of perfluoroundecanoic acid [PFUnDA, (CF)_10_], **UiO-68-SSeS** significantly outperforms **UiO-68-TzSeTz**, achieving a removal efficiency of 91% compared to 35%, respectively. Under the same conditions, UiO-68 generally exhibits higher removal efficiencies, particularly for long-chain PFAS (Figure 6). The better performance can be ascribed to the higher amount of TFA in UiO-68 (2.8 molecules per formula unit) with respect to those of **UiO-68-SSeS** and **UiO-68-TzSeTz** (2.1 and 2.4 molecules per formula unit, respectively), which is expected to enhance fluorophilic interactions with PFAS molecules.^51^ For shorter chains (PFHxS and PFOA), our MIXMOFs show better adsorption capacity than UiO-68, possibly because in this case the strongest host–guest interactions at work are between the polar –COOH/-SO_3_H groups present in PFHxS and PFOA and the (N,S,Se) heteroatoms present in SSeS^2–^ and TzSeTz^2–^. Given that all our MOFs contain TFA in the defects, a possible water contamination deriving from TFA release in solution is to be excluded, since the linker exchange process in zirconium MOFs is well-known to occur at temperatures higher than ambient (typically above 353 K).^29,39^ The higher removal efficiency observed for PFOS compared to PFOA, despite their identical perfluorinated chain length, is consistent with well-established trends reported in the literature, where sulfonate PFAS generally exhibit stronger adsorption affinity than their carboxylate analogues. This behavior has also been observed in MOFs, where PFOS shows enhanced uptake relative to PFOA due to stronger hydrophobic and fluorophilic interactions with the pore environment and framework surface chemistry.^52^ In contrast, carboxylated PFAS are comparatively more hydrophilic and tend to exhibit weaker interactions with adsorbent surfaces, resulting in reduced partitioning into MOF pores. As a consequence, sulfonated PFAS typically display enhanced affinity toward hydrophobic and fluorophilic domains of MOF-based adsorbents, even at equal chain length. Overall, when considering the cumulative adsorption capacity expressed as micrograms of PFAS removed per gram of sorbent, **UiO-68-SSeS** exhibits a slightly higher performance relative to **UiO-68-TzSeTz** (0.90 vs 0.82 μg/g). Both materials preferentially adsorb long-chain PFAS, with **UiO-68-SSeS** displaying enhanced affinity toward the longest-chain species, likely reflecting differences in the textural properties (§3.4), pore walls decoration, and insurgence of specific preferential host–guest interactions. The higher adsorption performances toward PFAS of zirconium-based MOFs has been attributed to the hydrophobic nature of the pollutant that matches well with that of the MOF pores^53^ and to the fluorophilic interactions between the PFAS molecule and the fluorine atoms present in the MOF pores (in this specific case, coming from the TFA units on the defects).^52,54^ Comparison with literature results is not feasible because of the different initial concentrations of spiked water. Previous works reported high removal efficiencies using highly concentrated feed solutions, typically with initial PFAS concentrations exceeding 100 ppm,^55–57^ conditions that are not representative of PFAS levels commonly found in contaminated waters. At aqueous PFAS concentration in the 10 ÷ 1000 ppb range (i.e., around or above their critical micelle concentration), PFAS begin to self-assemble into micelles or larger aggregates, forming localized clusters in solution and at interfaces.^58,59^ Such (hemi)­micelle or multilayer formation on more hydrophobic sorbent surfaces can enhance PFAS sorption and apparent removal capacity.^60^ In contrast, the adsorption experiments reported herein were conducted at PFAS concentrations consistent with those typically detected in contaminated water destinated to potabilization.^49,50^ It should be noted that the variability observed in the removal efficiencies, particularly for long-chain PFAS, is consistent with the analytical uncertainty typically associated with PFAS quantification, where relative standard deviations in the range of 15–30% are commonly reported.^61^

**Figure 6:**
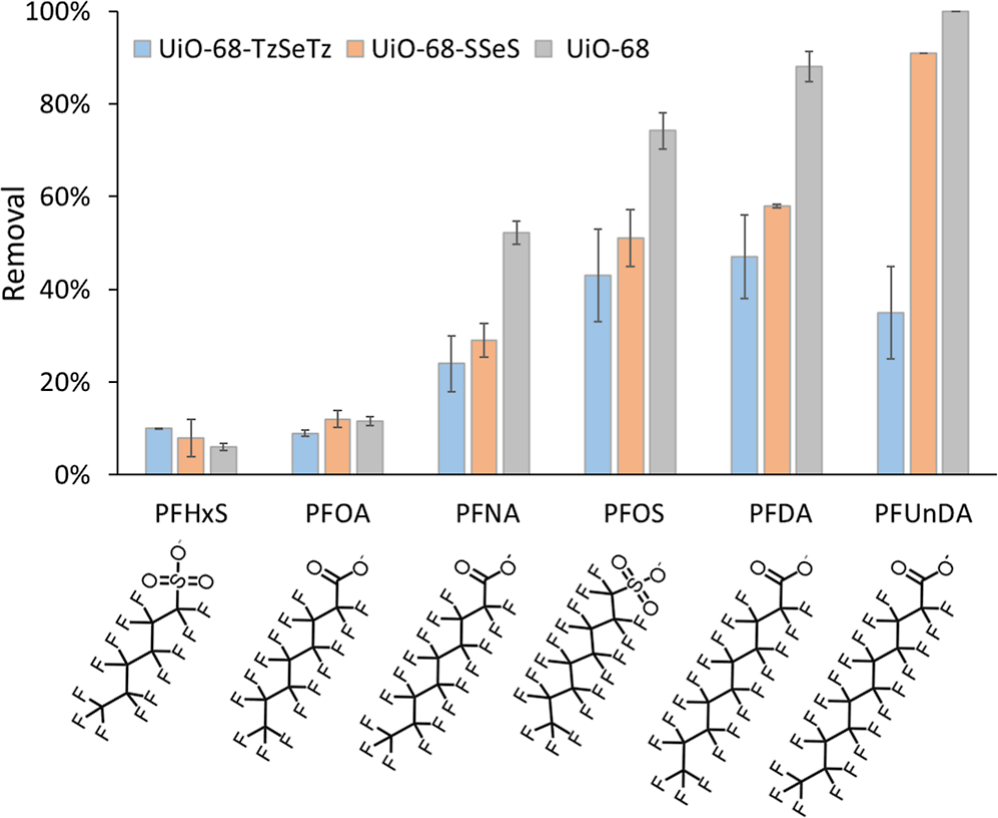
PFAS percentage removal from a tap water solution 0.1–3 μg/L in each PFAS by **UiO-68-TzSeTz** (blue) compared to **UiO-68-SSeS** (orange) and UiO-68 (gray).

## 4 Conclusions

In this study, two UiO-68-type mixed-linker Zr^IV^ MOFs incorporating selenophene-based heteroaromatic linkers have been successfully designed and thoroughly characterized. Powder diffraction structural characterization revealed the presence of missing-linker defects, resulting in modified pore environments but still significant accessible surface areas. From a functional standpoint, the incorporation of selenophene-based linkers markedly alters the optical response of the parent MOF, producing strong green luminescence and extending the excitation into the visible range, as confirmed by steady-state and confocal fluorescence lifetime imaging. These findings highlight the potential of heteroaromatic linkers to impart additional photophysical functionality to Zr-based MOFs without compromising their structural integrity. Importantly, both **UiO-68-SSeS** and **UiO-68-TzSeTz** demonstrate good adsorption of long-chain PFAS from tap water at environmentally relevant concentrations in the parts per billion range, with **UiO-68-SSeS** exhibiting particularly high affinity for the longest-chain species investigated. The observed chain-length-dependent adsorption underscores the role of textural properties, pore chemistry, linker polarizability, and host–guest interactions in governing PFAS uptake. Overall, this work illustrates how rational linker engineering and a mixed-linker MOF choice can be exploited to fine-tune the structural, optical, and adsorption properties of MOFs, providing a versatile platform for the development of multifunctional materials aimed at advanced water remediation. Moreover, the Zr^IV^ MOF adsorption properties combined with their fluorescence represent an important tool toward PFAS monitoring applications. Studies in this direction are currently ongoing in our laboratories, with the aim of preparing better-performing MOFs for a combined PFAS luminescence sensing and adsorption dual activity.

## Supplementary Material











## Notes

Na2SSeS (CIF)
PPN2TzSeTz (CIF)
Crystal structure determination from single-crystal X-ray diffraction of H_2_SSeS and H_2_TzSeTz; additional characterization data of UiO-68, **UiO-68-SSeS**, and **UiO-68-TzSeTz**: IR spectra; TG-MS traces; Rietveld refinement plots, pore size distribution, linkers quantification through ^1^H and ^19^F NMR analysis of the digested samples; water stability of **UiO-68-SSeS** and **UiO-68-TzSeTz**; confocal images and spectra of UiO-68 and **UiO-68-TzSeTz**; fluorescence lifetime of UiO-68; and details of the UPLC-MS/MS analysis (PDF)
UiO-68-SSeS (CIF)
UiO-68-TzSeTz (CIF)
