# Supplementary material for: Metal–Organic Frameworks Built with Selenophene-Based Trimers for Perfluoroalkyl Substances Removal from Drinking Water
Source: Chem Mater. 2026 Jul 10;38(14):7159–71. doi: 10.1021/acs.chemmater.6c00594 (PMC13435745; doi:10.1021/acs.chemmater.6c00594)
Supplement: Supplementary file 1 [file cm6c00594_si_003.pdf]

Supporting Information

Metal-Organic Frameworks Built with Selenophene-Based Trimers for Perfluoroalkyl Substances Removal from Drinking Water

Giulio Bicchierai,[a,†] Andrea Trifoglio,[b,c,†] Laura Favaretto,[b] Daniele Evandri,[b,c] Sara Khaliha,[b] Ilse Manet,[b] Lidia Armelao,[d,e] Gregorio Bottaro,[f,g] Anna Mauri,[h] Simona Galli,[h,g] Giuliano Giambastiani,[i,a,g] Manuela Melucci*[b] and Andrea Rossin*[a,g]*

[a] CNR ICCOM, Istituto di Chimica dei Composti Organometallici, Via Madonna del Piano 10, 50019 Sesto Fiorentino (Firenze), Italy. E-mail: andrea.rossin@cnr.it

[b] CNR ISOF, Istituto per la Sintesi Organica e la Fotoreattività, Via P. Gobetti 101, I-40129 Bologna, Italy. E-mail: manuela.melucci@cnr.it

[c] Dipartimento di Chimica 'G. Ciamician', Alma Mater Studiorum – Università di Bologna, Via Selmi 2, 40126 Bologna, Italy

[d] Dipartimento di Scienze Chimiche (DiSC), Università di Padova, via F. Marzolo 1, 35131 Padova, Italy

[e] Dipartimento di Scienze Chimiche e Tecnologie dei Materiali (DSCTM), Consiglio Nazionale delle Ricerche (CNR), Piazzale A. Moro 7, 00185 Roma, Italy

[f] CNR-ICMATE, Istituto di Chimica della Materia Condensata e di Tecnologie per l'Energia, Via F. Marzolo 1, 35131 Padova, Italy

[g] Consorzio Interuniversitario per la Scienza e Tecnologia dei Materiali (INSTM), Via Giusti 9, 50121 Firenze, Italy.

[h] Dipartimento di Scienza e Alta Tecnologia, Università degli Studi dell'Insubria, Via Valleggio 9, 22100 Como, Italy. E-mail: simona.galli@uninsubria.it

[i] Dipartimento di Chimica "Ugo Schiff", Università di Firenze, Via della Lastruccia 3-13, Sesto Fiorentino (Firenze), 50019, Italy.

(† These authors contributed equally to this work)

Single-crystal X-ray diffraction analysis

Single-crystal X-ray diffraction data for **$Na_2SSeS\cdot(H_2O)$** and **$(PPN)_2TzSeTz\cdot6.6(H_2O)$** were collected at ambient (T = 298 K) or low (T = 219 K) temperature, respectively, on a Bruker APEX-II CCD diffractometer equipped with a sealed X-ray source (Cu Kα, λ = 1.5418 Å). Data were corrected for Lorentz and polarization effects, as well as for absorption using the software SADABS.[1] Direct methods, as implemented in Sir2014,[2] were used to solve the crystal and molecular structure. Structure refinement was then performed by full-matrix least-squares against F^2, as implemented in SHELXL-2018/2.[3] Most of the non-hydrogen atoms were refined anisotropically. All the hydrogen atoms of the organic cations were fixed in calculated positions (adopting the riding model) and refined isotropically with $U_{iso} = 1.2U_{eq}(C)$ (where C = carbon atom to which the H atom is bound). When feasible, the water molecule oxygen atoms were refined anisotropically and their hydrogen atoms were located based on the residual electron density maps and refined isotropically with $U_{iso} = 1.5U_{eq}(O)$ (where O = oxygen atom to which the H atom is bound). In the case of **$Na_2SSeS\cdot(H_2O)$**, the electron density of the coordinated water molecule appeared rather smeared, suggesting positional disorder. The latter was modelled through two oxygen atoms (O5A and O5B), fixing their site occupation factor (s.o.f.) to 0.5 and imposing the coincidence of their isotropic thermal parameter. No hydrogen atoms were attributed to O5A or O5B. As for **$(PPN)_2TzSeTz\cdot6.6(H_2O)$**, The smeared electron density ascribed to two clathrated water molecules was modelled, admitting positional disorder, by two oxygen atoms per molecule (O10A and O10B; O11A and O11B) with refined and correlated s.o.f.s [x and (1-x), respectively; (1-x) and x, respectively, given the proximity of O10A and O11A], and refined, isotropic thermal parameters. No hydrogen atoms were added to these atoms. The s.o.f. of atom O8, oxygen atom of a clathrated water molecule in too close proximity to O11B, was set to (1-x). No hydrogen atoms were added to O8. Figures S1 and S2 were produced with the program *Mercury 2026.1.1*.[4] CCDC 2531329 and 2531330 contain the supplementary crystallographic data for this paper. These data can be obtained free of charge from The Cambridge Crystallographic Data Centre *via* https://www.ccdc.cam.ac.uk/structures/. Tables S1 and S2 collect the main experimental details and crystallographic data.

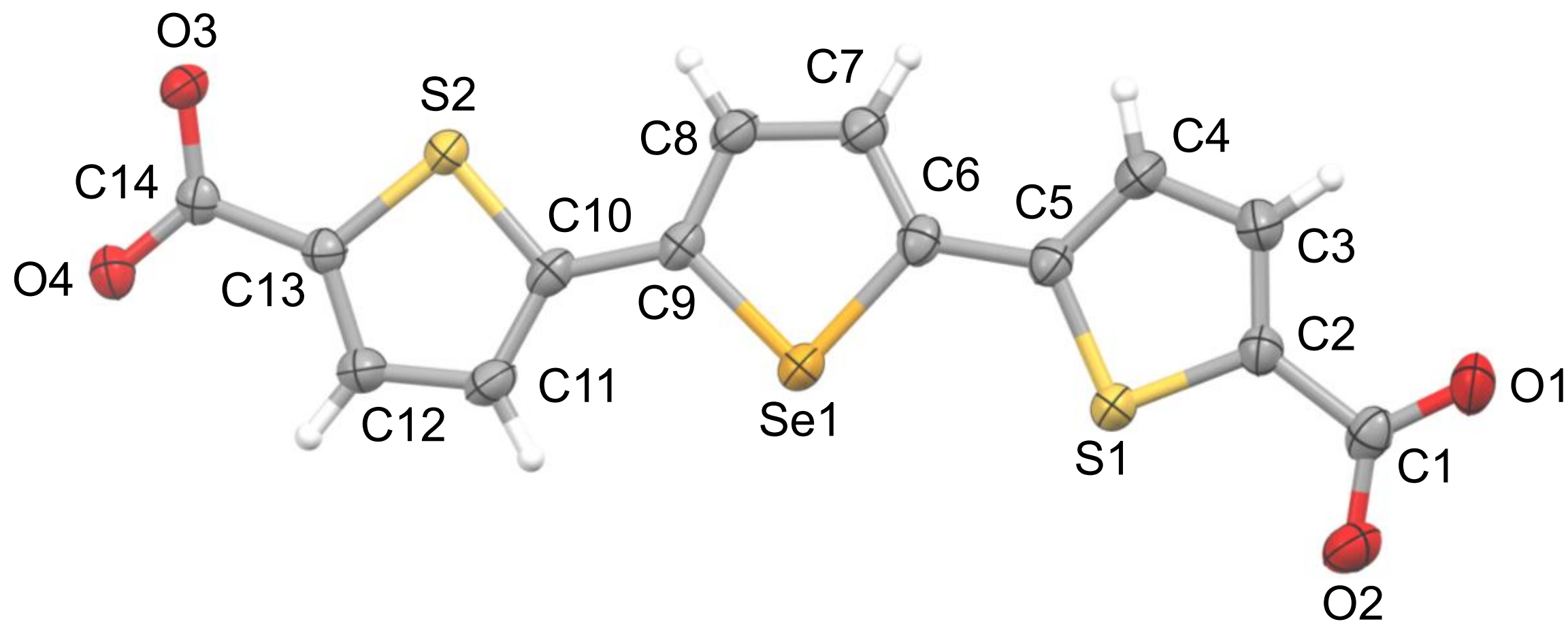

Figure S1. ORTEP drawing at 50% probability level of the anionic moiety in **Na_2SSeS·(H_2O)**.

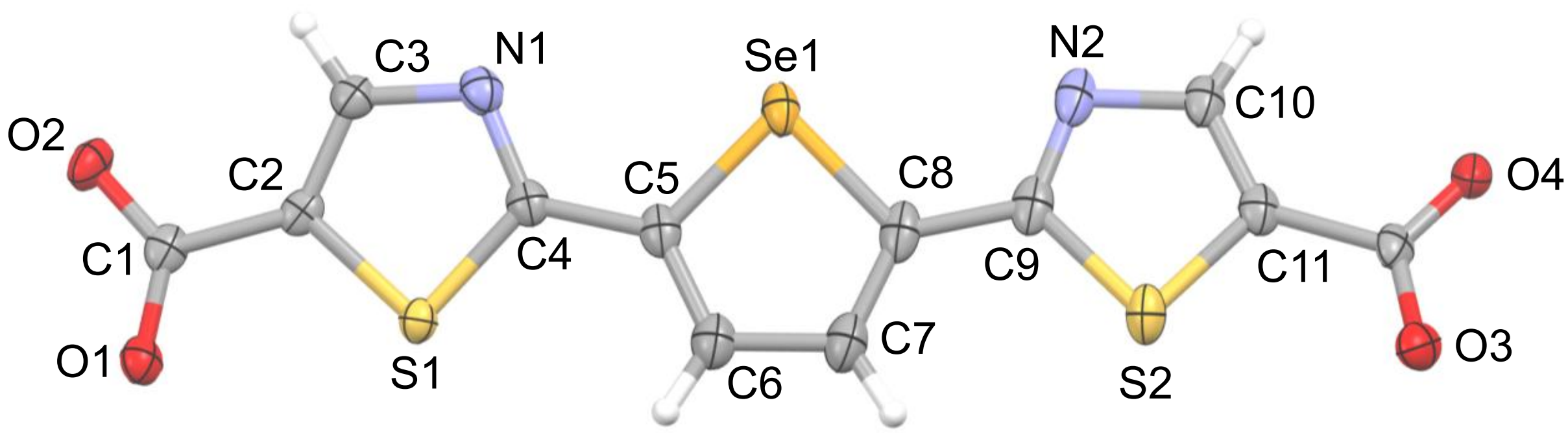

Figure S2. ORTEP drawing at 50% probability level of the anionic moiety in **(PPN)$_2$TzSeTz·6.6(H_2O)**.

Table S1. Main crystallographic data and structure refinement details for **Na_2SSeS·(H_2O)**.

Formula	C_{14} H_6 Na_2 O_5 S_2 Se
M [g mol^{-1}]	443.25
T [K]	298
Colour, habit	Yellow-bronze, prism
Size [mm^3]	0.200 × 0.060 × 0.060
Crystallographic system	Orthorhombic
Space group	*Pccn*
Z	8
a [Å]	14.9341(5)
b [Å]	33.1843(11)
c [Å]	6.1283(2)
V [Å^3]	3037.05(17)
F(000)	1744
μ (Cu Kα) [mm^{-1}]	6.718
D_{calc} [g cm^{-3}]	1.939
θ range for collection [°]	2.663 ÷ 68.313
h, k, l ranges for collection	$-16 \leq h \leq 17$, $-39 \leq k \leq 39$, $-7 \leq l \leq 7$
Measured reflns	29504
Unique reflns	2789
Completeness [%]	99.9 (to θ = 67.679°)
R_{int}	0.0620
Data / restraints / parameters	2789 / 0 / 215
R1, wR2 [$I > 2\sigma(I)$]	0.030, 0.072
R1, wR2 (all data)	0.035, 0.075
Gof on F^2	1.145
Max peak, min hole [e Å^{-3}]	0.410, –0.659

Table S2. Main crystallographic data and structure refinement details for **$(PPN)_2$TzSeTz · 6.6(H_2O)**.

Formula[a]	$C_{84}H_{72}N_4O_{10.6}P_4S_2Se$
M [g mol^{-1}]	1573.54
T [K]	219
Colour, habit	Light yellow, prism
Size [mm^3]	0.14 × 0.14 × 0.19
Crystallographic system	Triclinic
Space group	P-1
Z	2
a [Å]	14.0595(9)
b [Å]	14.9808(9)
c [Å]	19.4706(12)
α [°]	78.082(3)
β [°]	81.669(3)
γ [°]	70.112(3)
V [Å^3]	3760.8(4)
F(000)	1648
μ (Cu Kα) [mm^{-1}]	2.524
D_{calc} [g cm^{-3}]	1.389
θ range for collection [°]	2.327 ÷ 68.619
h, k, l ranges for collection	$-16 \leq h \leq 16$, $-18 \leq k \leq 18$, $-23 \leq l \leq 23$
Measured reflns	129454
Unique reflns	13790
Completeness [%]	99.9 (to θ = 67.679°)
R_{int}	0.0727
Data / restraints / parameters	13790 / 0 / 978
R1, wR2 [$I > 2\sigma(I)$]	0.0401, 0.1029
R1, wR2 (all data)	0.0433, 0.1057
Gof on F^2	0.829
Max peak, min hole [e Å^{-3}]	0.550, –1.075

[a] For the actual value of oxygen and hydrogen atoms, deriving from the existence of vicariance or positional disorder affecting some water molecules, the reader is referred to the details of the crystal structure analysis in page S2 of this document.

Defects quantification in UiO-68 through combined ^{1}H and ^{19}F NMR spectroscopy of the digested solid

An accurate estimation of the amount of defects present in the home-made UiO-68 was performed using ^{1}H (τ = 5 s; 32 scans) and ^{19}F (τ = 10 s; 32 scans) NMR spectroscopy in the presence of the internal standard 2,6-difluorobenzoic acid (DFB, $C_7H_4F_2O_2$, FW = 158.1), following a methodology similar to that reported by Farha *et al.* in 2013.[5] 0.005 g of UiO-68 and 0.005 g of DFB were dissolved in 0.5 mL of D_2SO_4/D_2O (Sigma Aldrich). The moles of H_4TCPB and TFA were calculated from ^{1}H and ^{19}F signal integration against the standard, respectively, and taking into account the different number of ^{1}H or ^{19}F nuclei in the two molecules. The [H_4TCPB/TFA] molar ratio was then exploited to derive UiO-68 minimal formula.

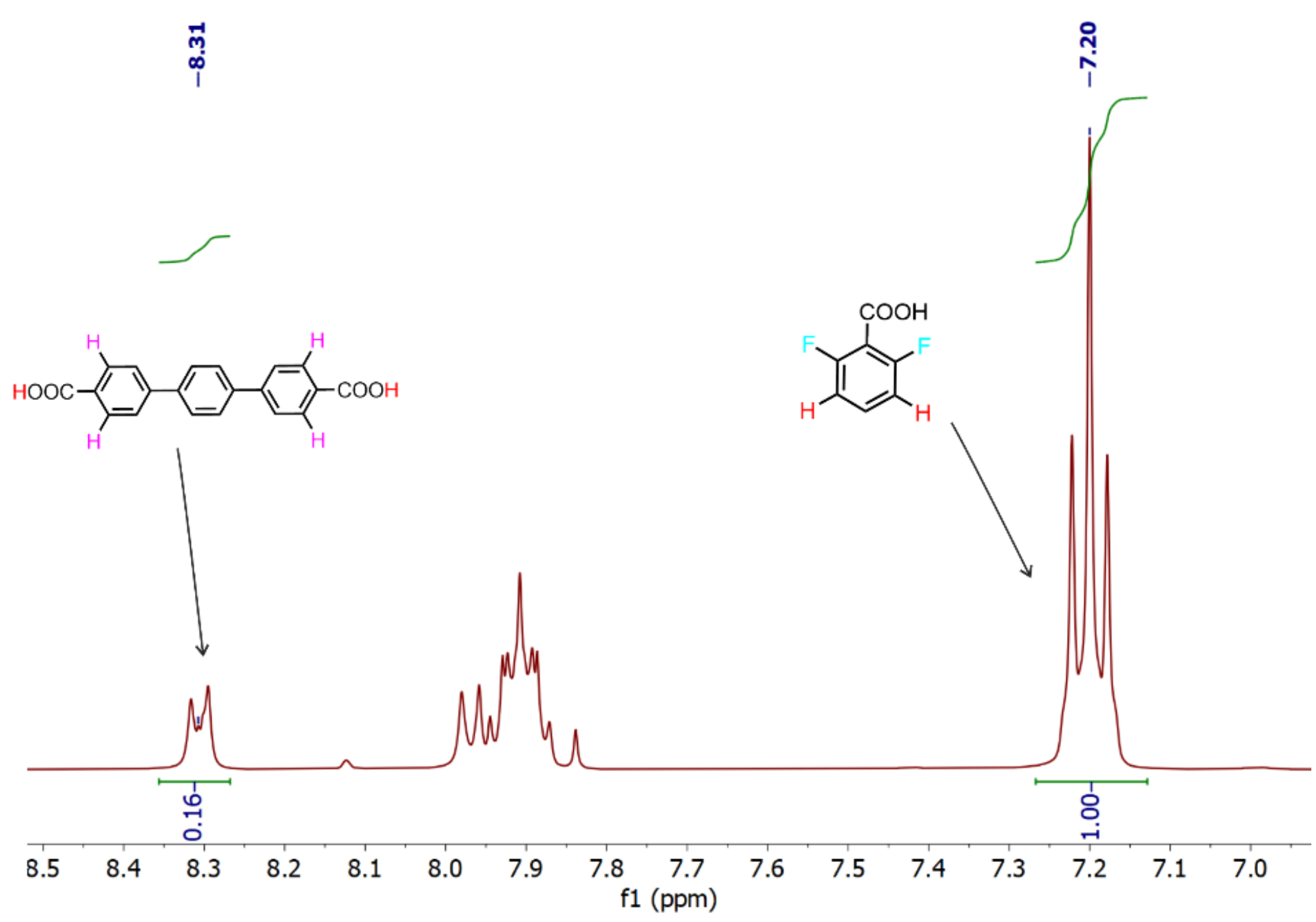

Figure S3. ^{1}H NMR spectrum (400 MHz, D_2SO_4/D_2O, T = 298 K) of digested UiO-68.

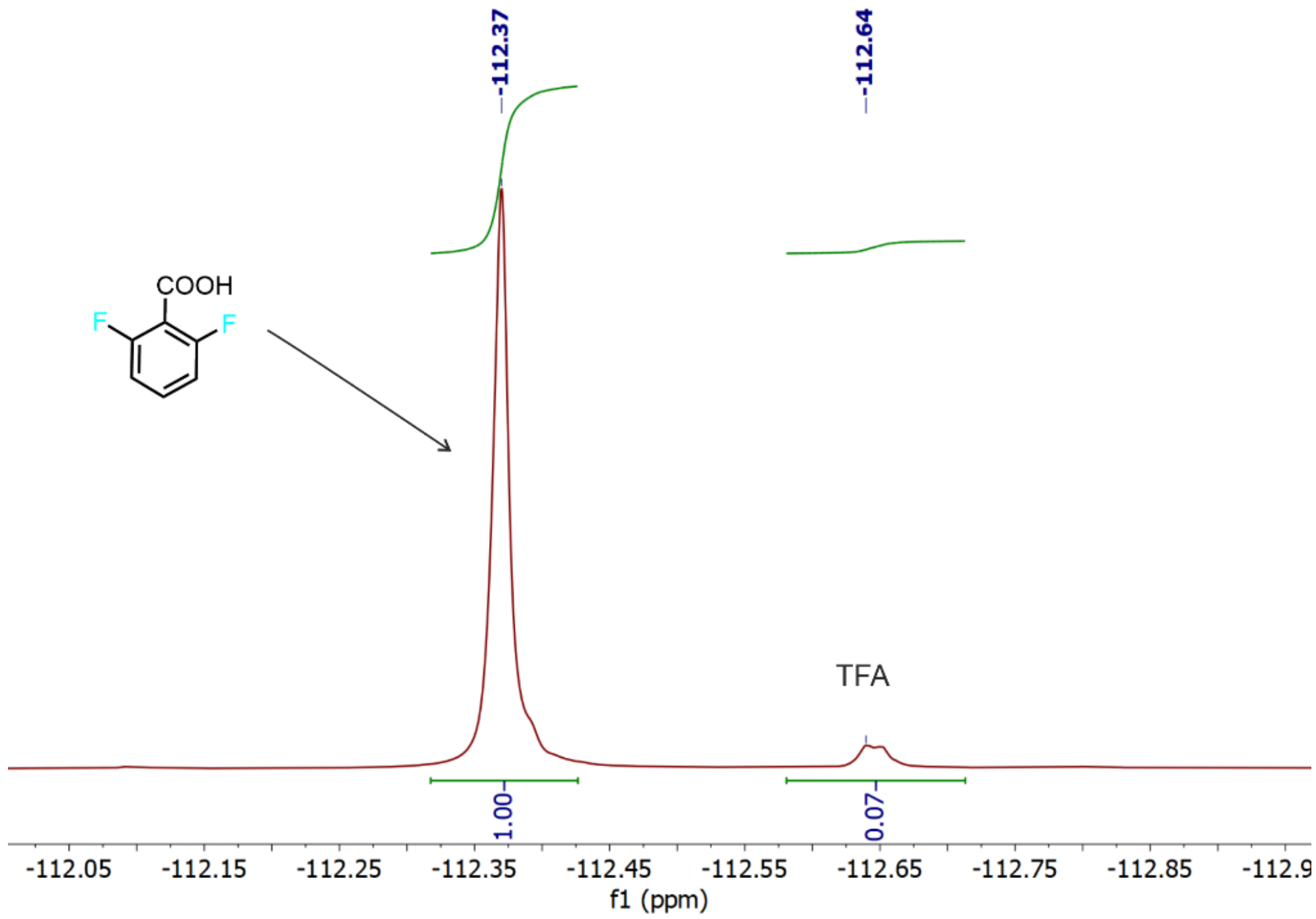

Figure S4. ^{19}F NMR spectrum (376 MHz, D_2SO_4/D_2O, T = 298 K) of digested UiO-68.

Mmol DFB = 5/158.1 = 0.032. From the ^{1}H NMR integral values: mmol H_4TCPB = (0.032 × 0.04)/0.5 = 0.0026; from the ^{19}F NMR integral values: mmol TFA = (0.032 × 0.023)/0.5 = 0.0015. Thus: [H_4TCPB/TFA] molar ratio ≈ 1.7. For a minimal formula $[Zr_6O_4(OH)_4(TPDC)_x(TFA)_{12-2x}]$, we obtain x = 4.6.

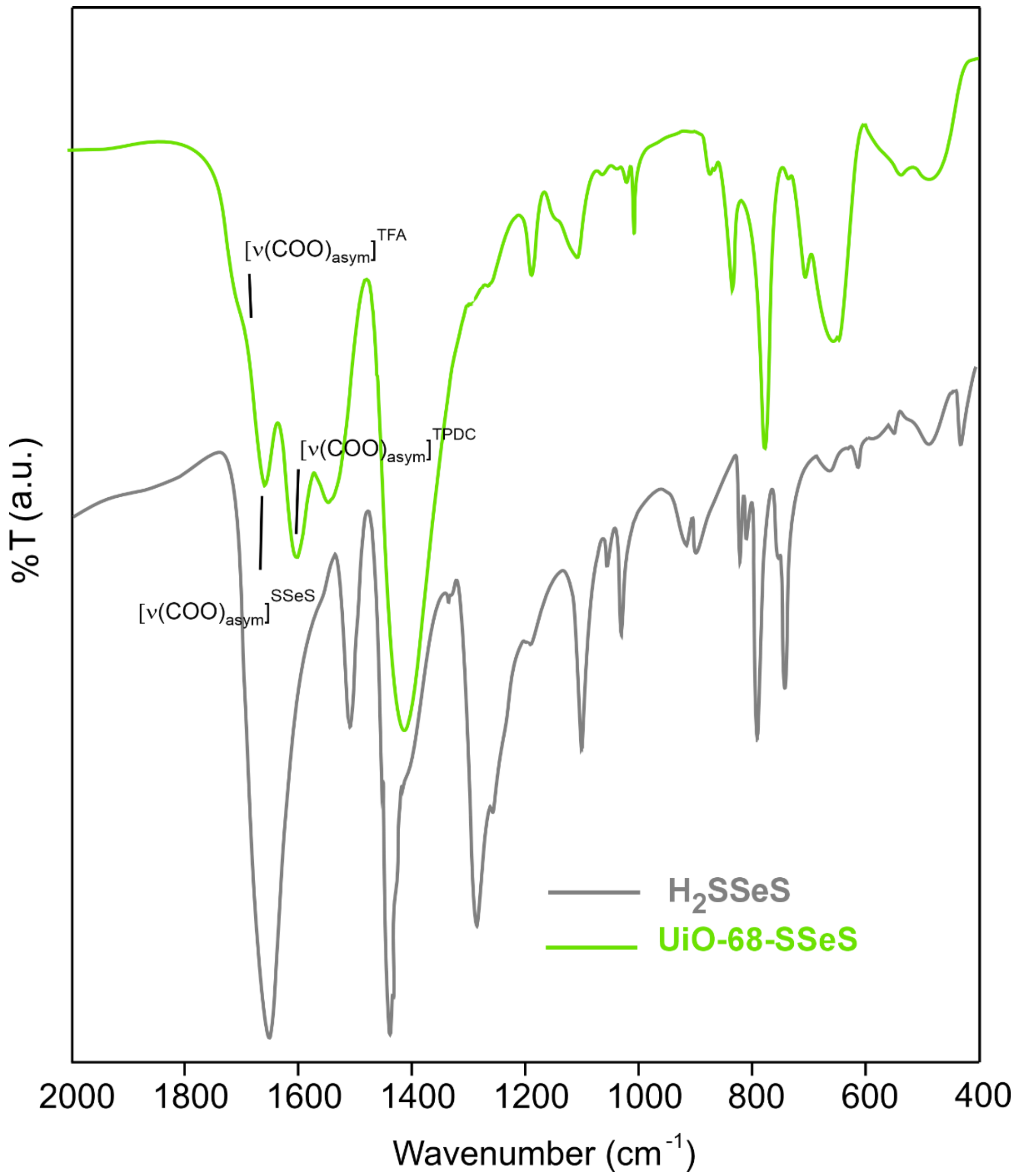

Figure S5. Most significant region of the infrared spectra (KBr, T = 298 K, 4000-400 cm^{-1}) of **UiO-68-SSeS** and its constitutive linker H_2SSeS at comparison.

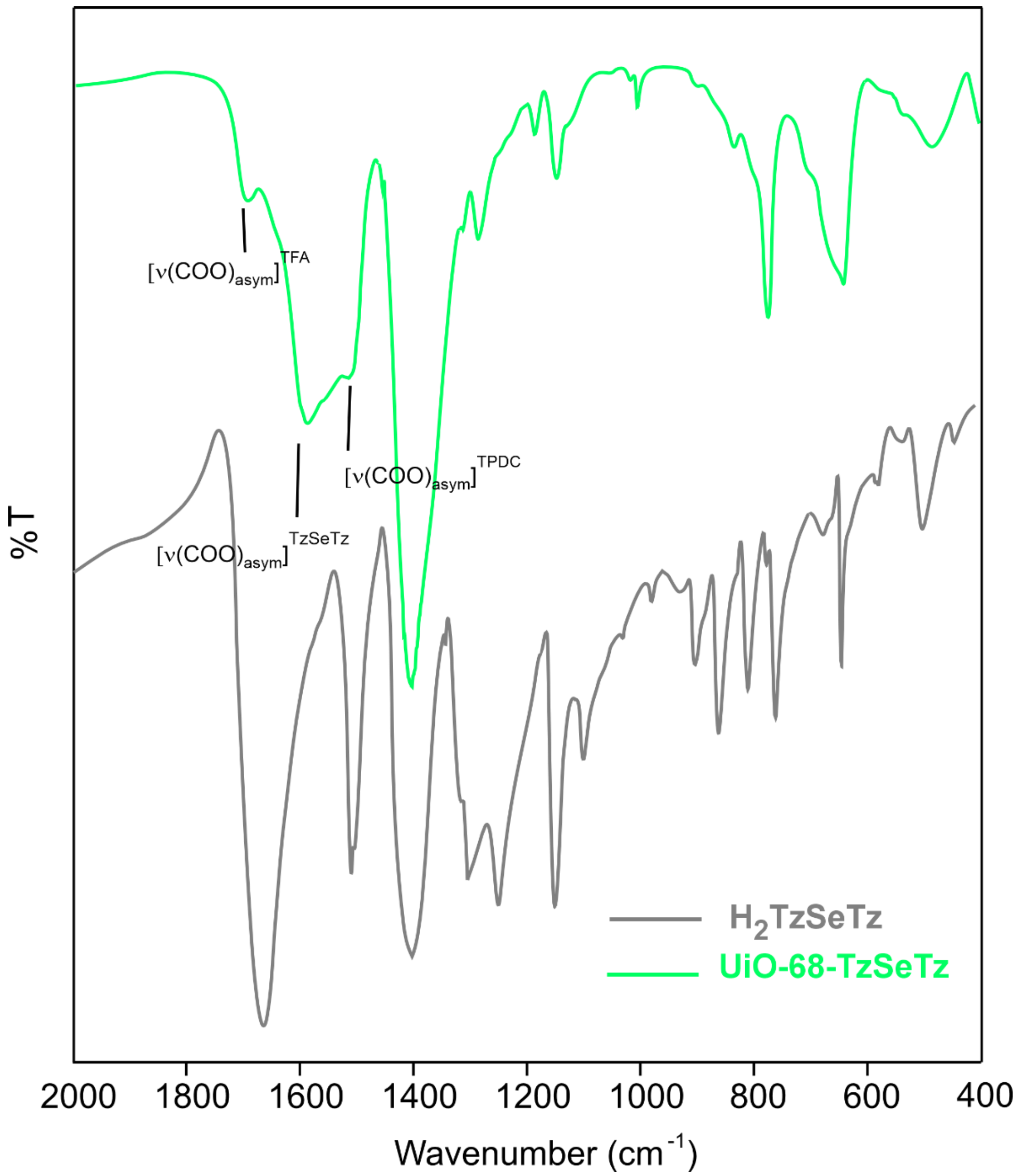

Figure S6. Most significant region of the infrared spectra (KBr, T = 298 K, 4000-400 cm^{-1}) of **UiO-68-TzSeTz** and its constitutive linker $H_2TzSeTz$ at comparison.

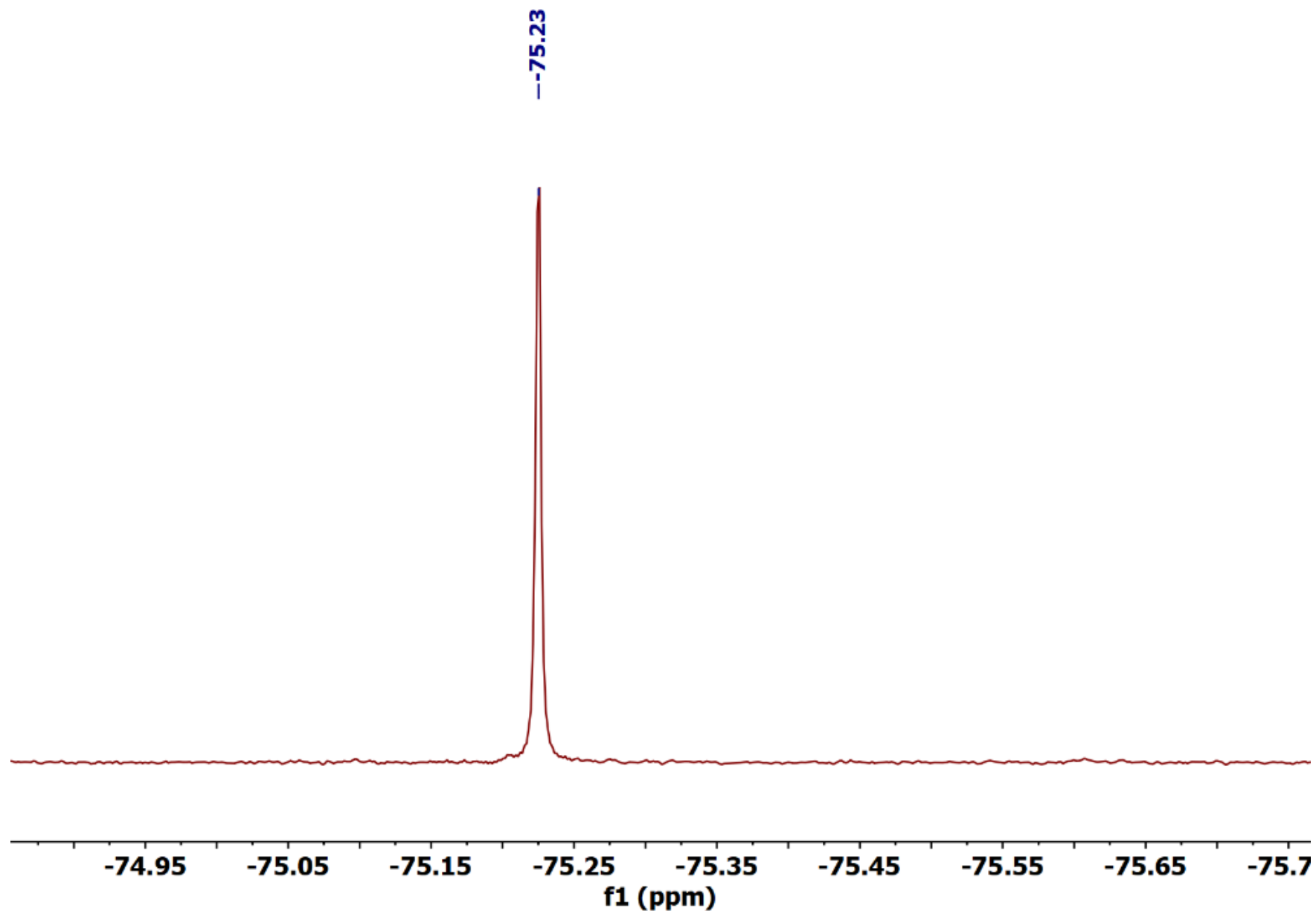

Figure S7. ^{19}F NMR spectrum (564 MHz, D_2SO_4/D_2O/DMSO-d_6, T = 298 K) of digested **UiO-68-SSeS**. The same spectrum was acquired on a digested sample of **UiO-68-TzSeTz**.

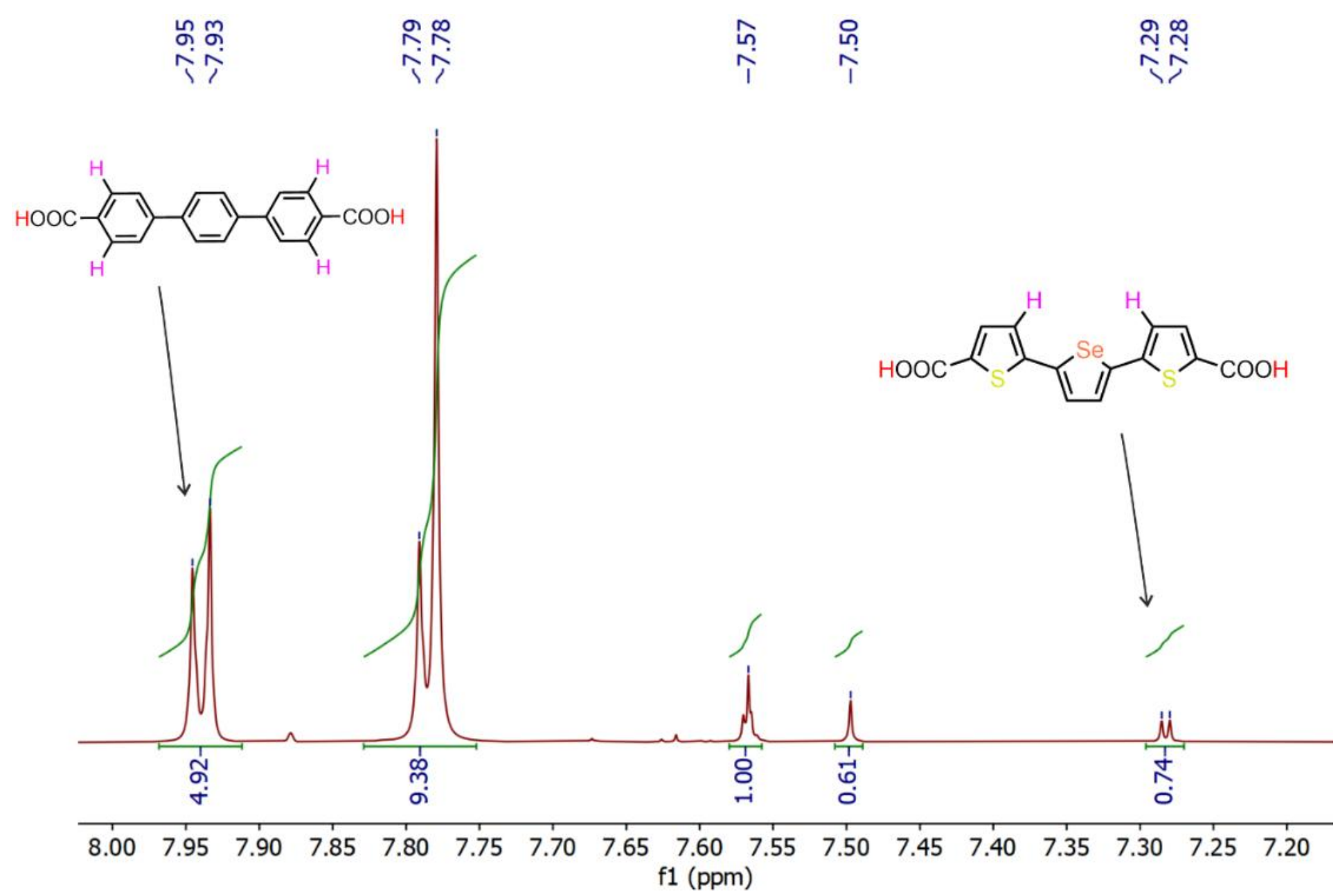

Figure S8. Magnification of the aromatic region used for the quantification of the ligands ratio from the ^{1}H NMR spectrum (400 MHz, D_2SO_4/D_2O/DMSO-d_6, T = 298 K) of digested **UiO-68-SSeS**.

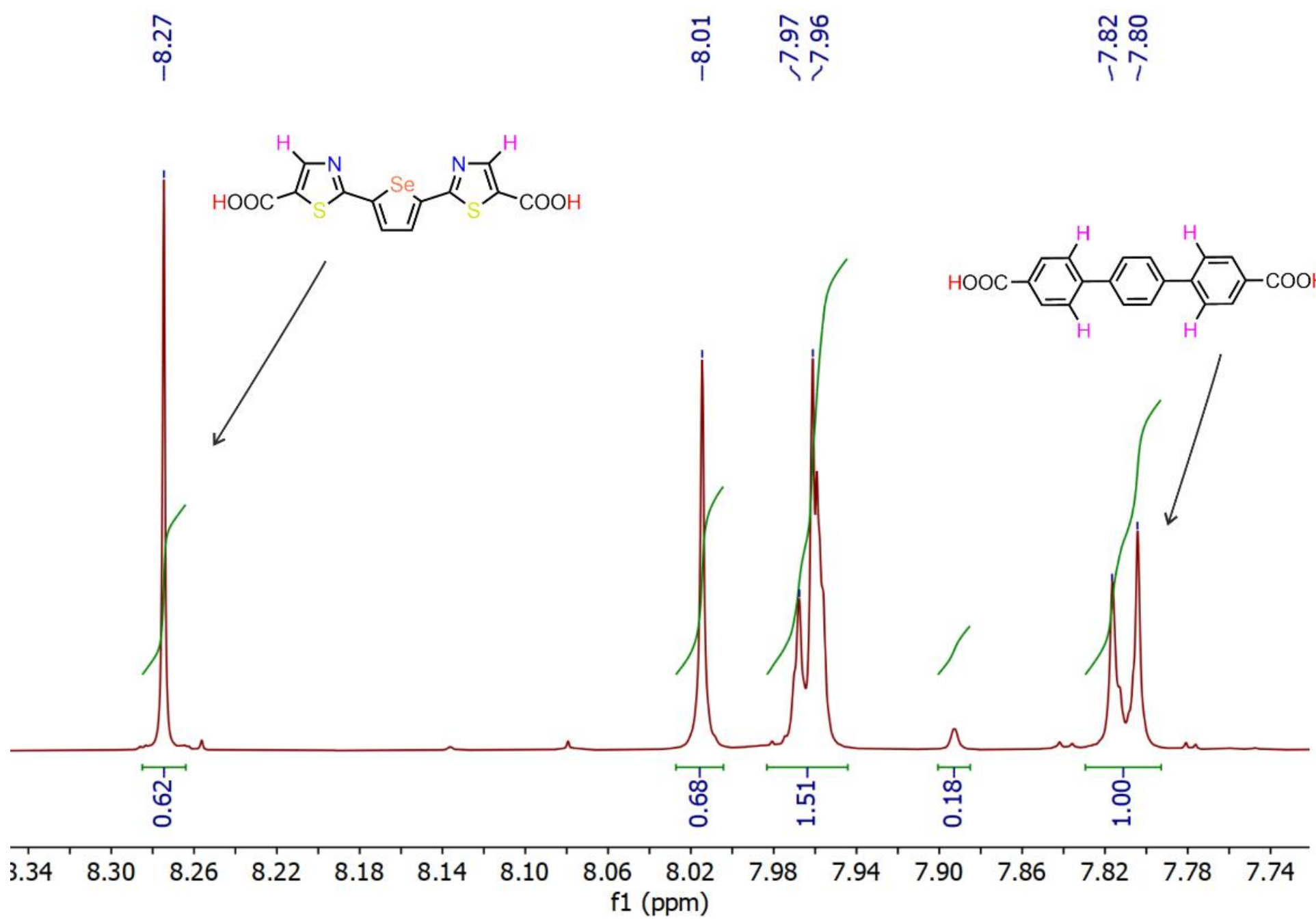

Figure S9. Magnification of the aromatic region used for the quantification of the ligands ratio from the ^{1}H NMR spectrum (400 MHz, D_2SO_4/D_2O/DMSO-d_6, T = 298 K) of digested **UiO-68-TzSeTz**.

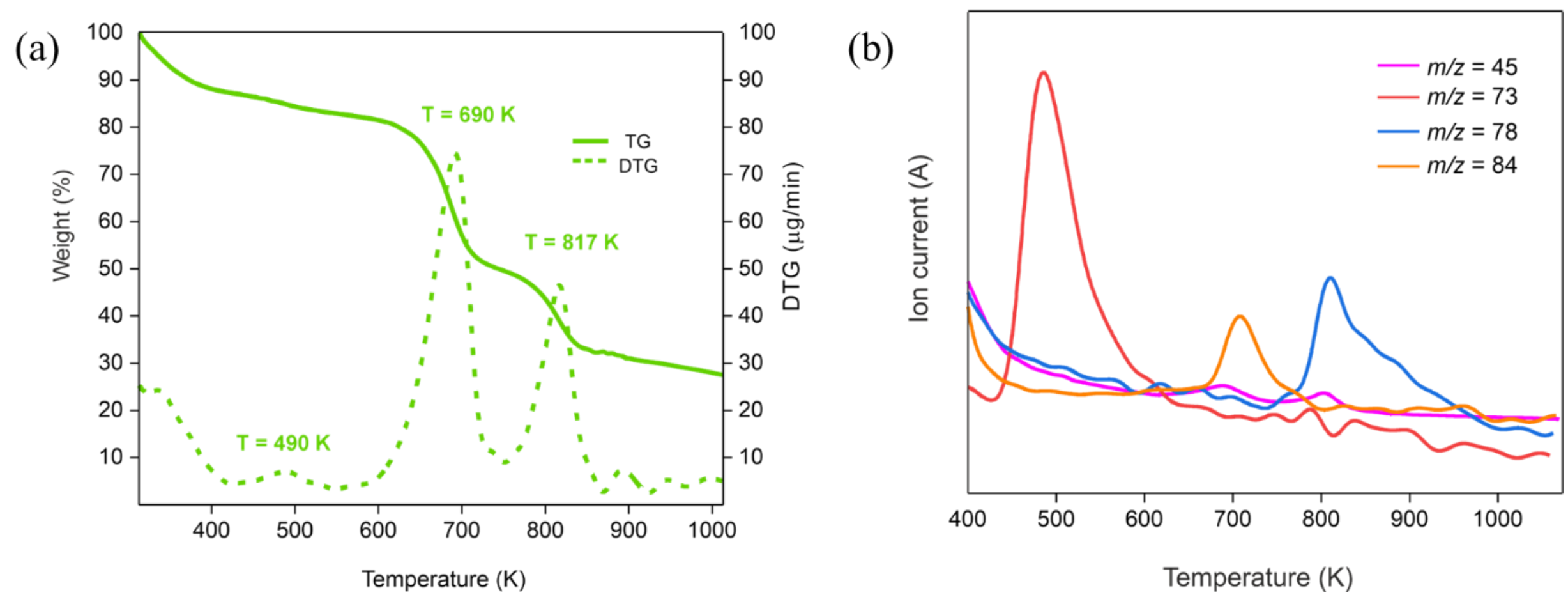

Figure S10. TG-DTG traces (a) and related mass spectrometry peaks (b) of **UiO-68-SSeS**.

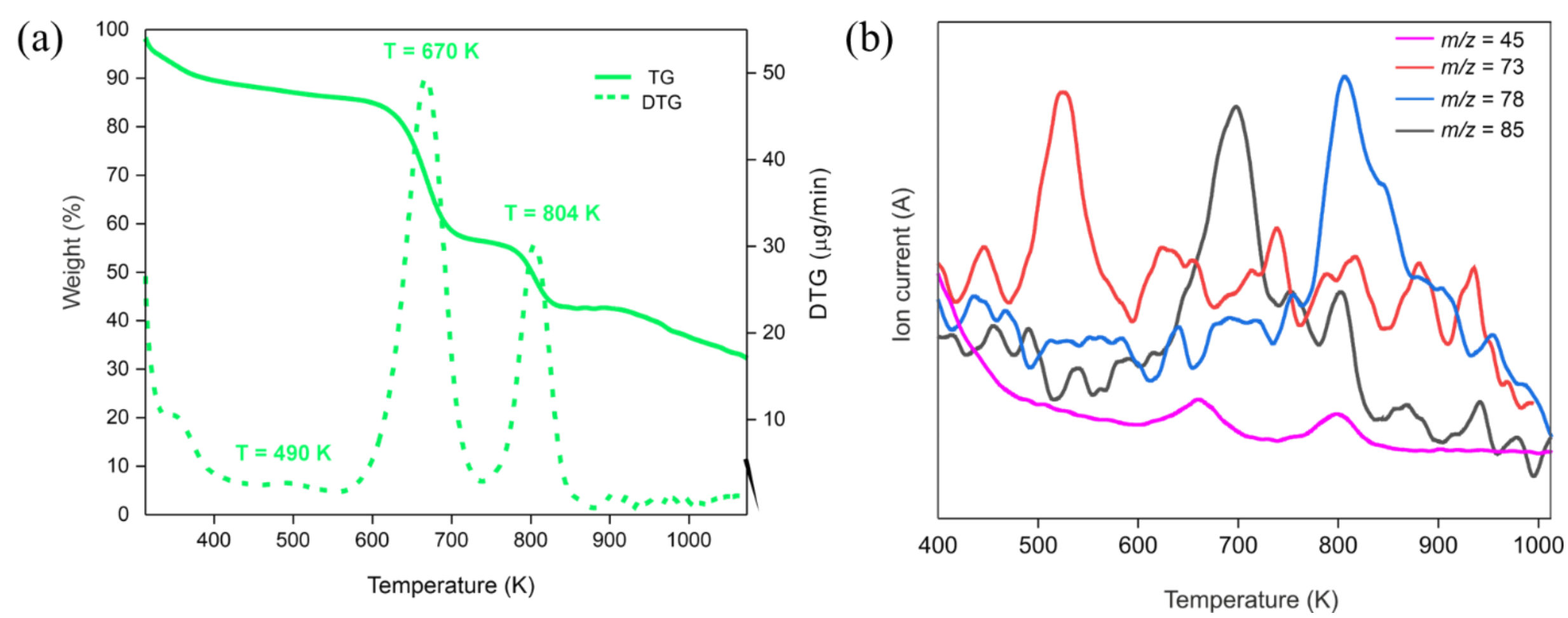

Figure S11. TG-DTG traces (a) and related mass spectrometry peaks (b) of **UiO-68-TzSeTz**.

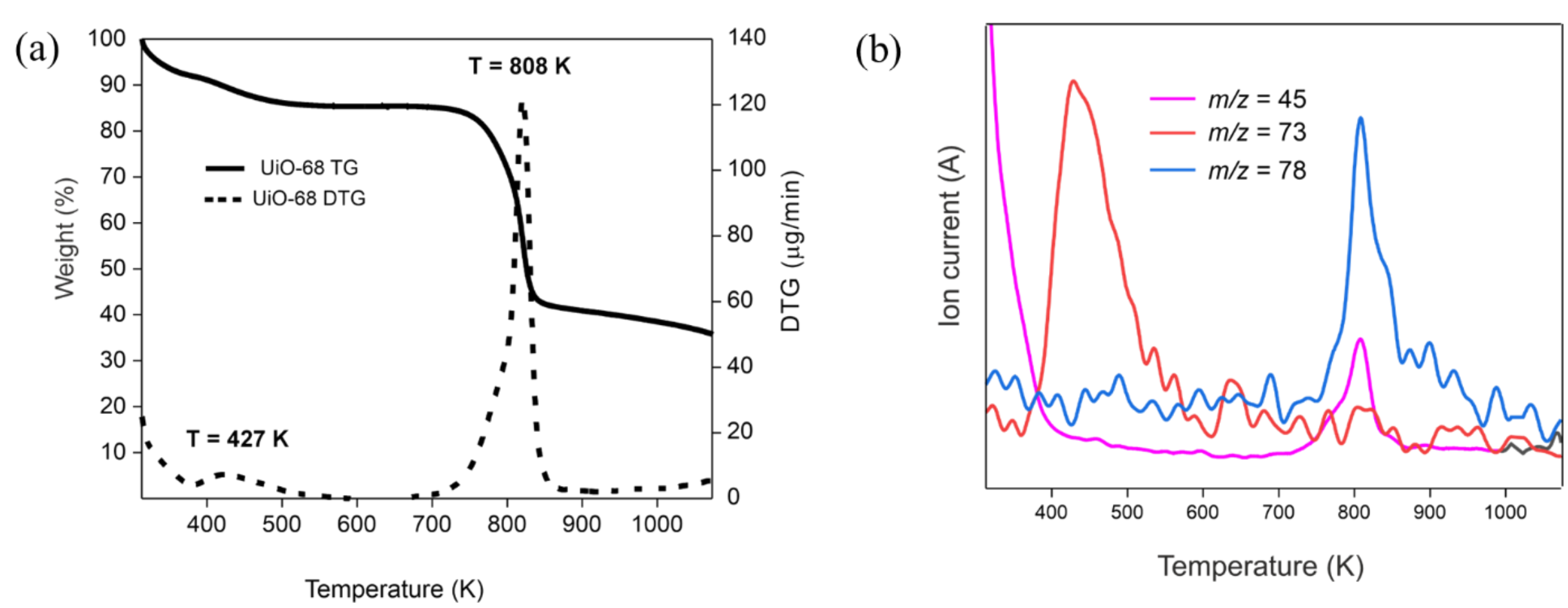

Figure S12. TG-DTG traces (a) and related mass spectrometry peaks (b) of UiO-68.

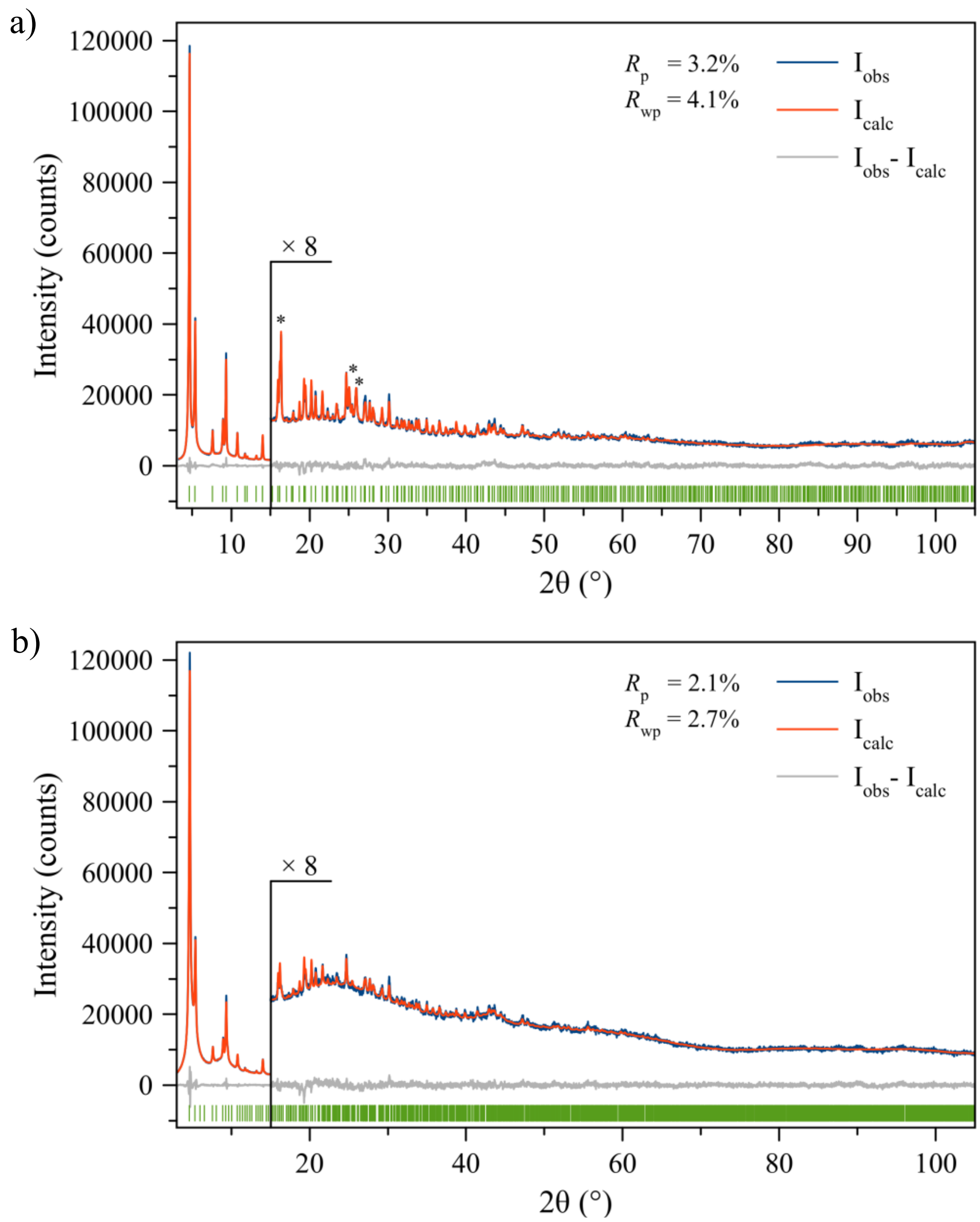

Figure S13. Graphical result of the final stage of the structure refinement, performed with the Rietveld method, on the PXRD data acquired on a) **UiO-68-SSeS** and b) **UiO-68-TzSeTz**. Blue, red and grey lines: observed, calculated and difference patterns, respectively. The portion above 15° has been magnified for clarity. The ticks at the bottom represent the position of the Bragg reflection maxima. Asterisks in a) indicate peaks attributed to a non-identified phase.

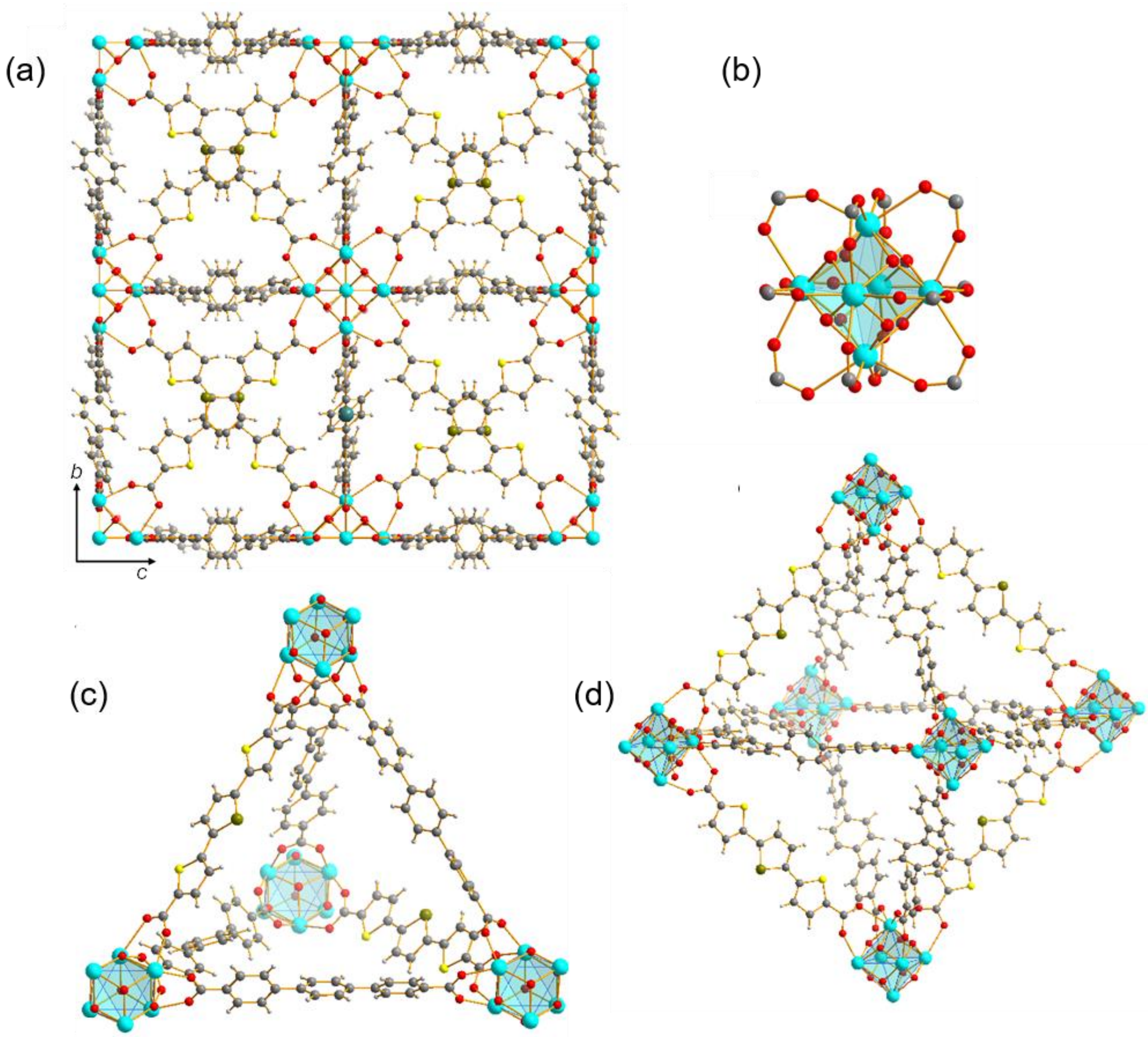

Figure S14. Representation of the crystal structure of **UiO-68-SSeS**: a) portion of the crystal structure viewed along one of the unit cell axes; b) the node; c) the tetrahedral cavity; d) the octahedral cavity. For simplicity, an ideal formula disregarding missing linker defects was adopted. Element colour code: C, dark grey, H, light grey; O, red; S, yellow; Se, gold; Zr, cyan.

Table S3. Angles between the plane of the carboxylate groups and the mean square plane of the carbocyclic or heterocyclic rings in **UiO-68-SSeS** and **UiO-68-TzSeTz** and empty volume, as retrieved at the end of the structural characterization performed with the Rietveld method using the software *Mercury*,[6] at comparison with the corresponding values in UiO-68 according to Manna[7] and Schaate.[8] For the sake of completeness, the table also includes the X-ray diffraction data acquisition temperature.

	**UiO-68**[7]	**UiO-68**[8]	**UiO-68-SSeS**	**UiO-68-TzSeTz**	
	$TPDC^{2-}$	$TPDC^{2-}$	$TPDC^{2-}$	$TPDC^{2-}$	$TzSeTz^{2-}$
**T (K)**	100	173	298	298	
**Central ring (°)**	0	53.5	57.1	0.66	55.6
**Lateral rings (°)**	0	0	24.2	70.9/20.5[a]	8.3/3.9[a]
**Empty V (%)**	76.8	74.7	67.1	71.5	

[a] Due to the lower symmetry of space group *Pa*-3, in **UiO-68-TzSeTz** there are two crystallographically independent lateral rings.

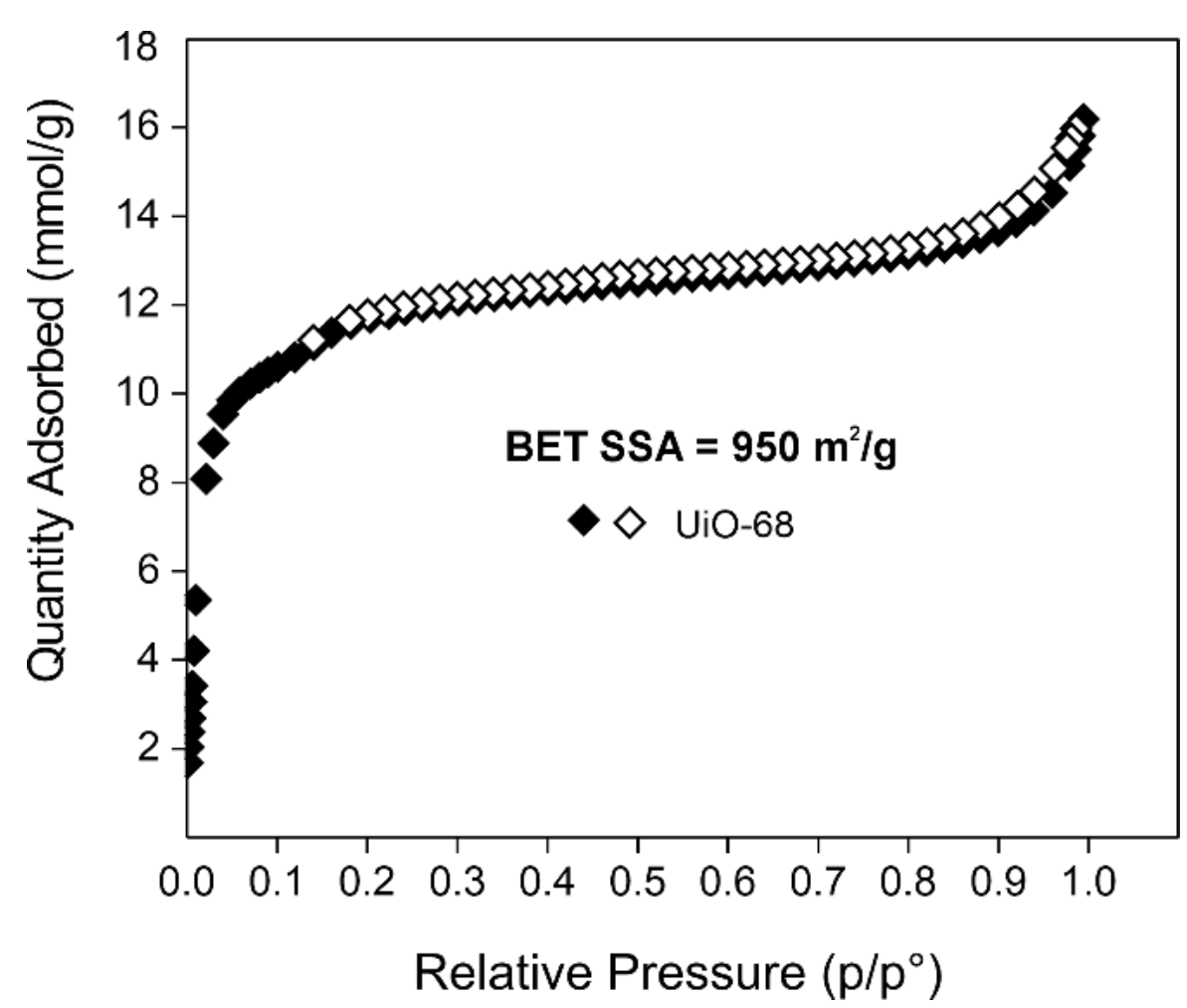

Figure S15. N_2 adsorption isotherms collected at T = 77 K on the home-made UiO-68, starting material used for the synthesis of the MIXMOFs presented in this work. Empty symbols refer to the desorption branch.

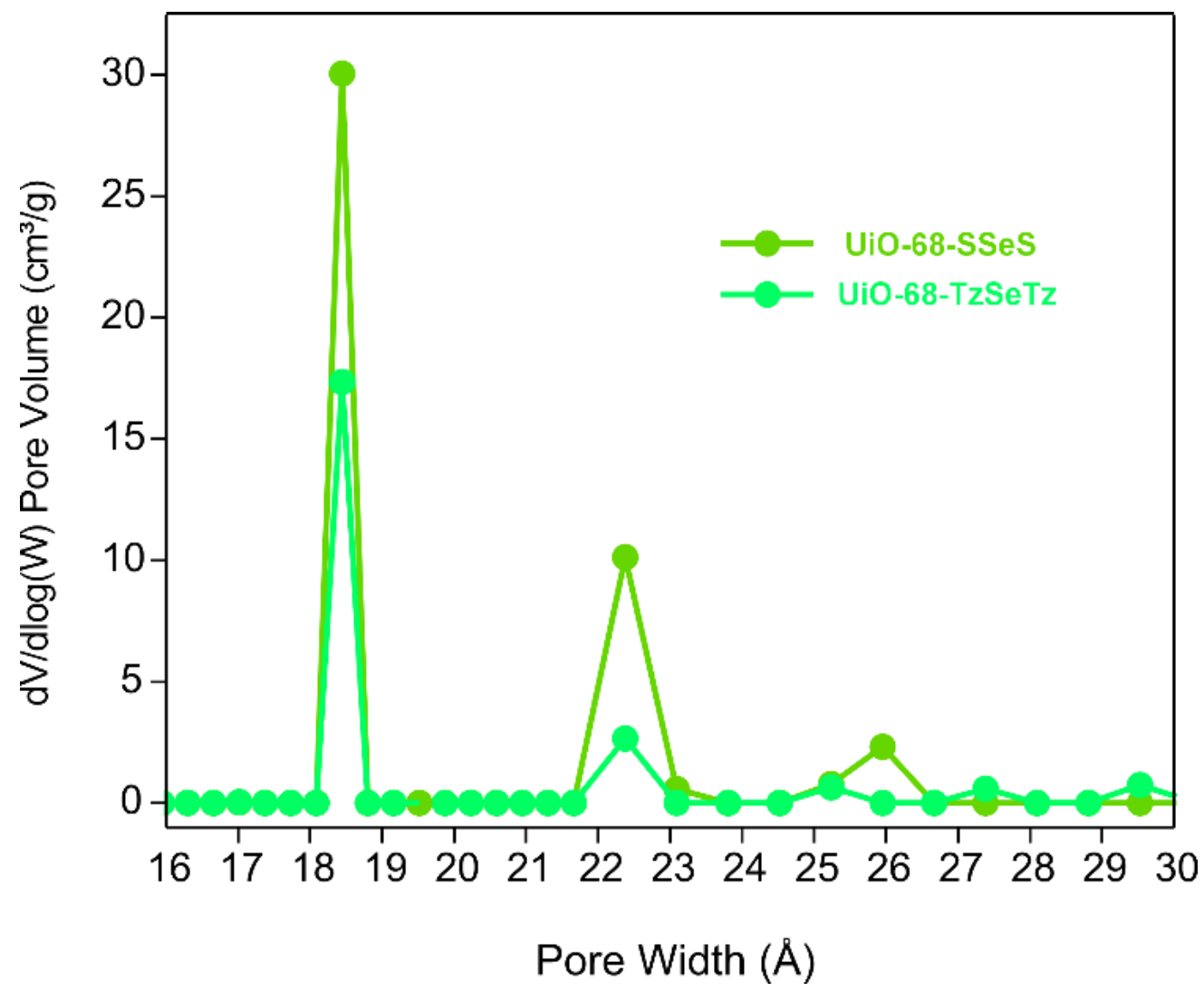

Figure S16. Micropore size distribution (NLDFT method – Tarazona approximation, cylindrical pore shape) of **UiO-68-SSeS** and **UiO-68-TzSeTz** at comparison.

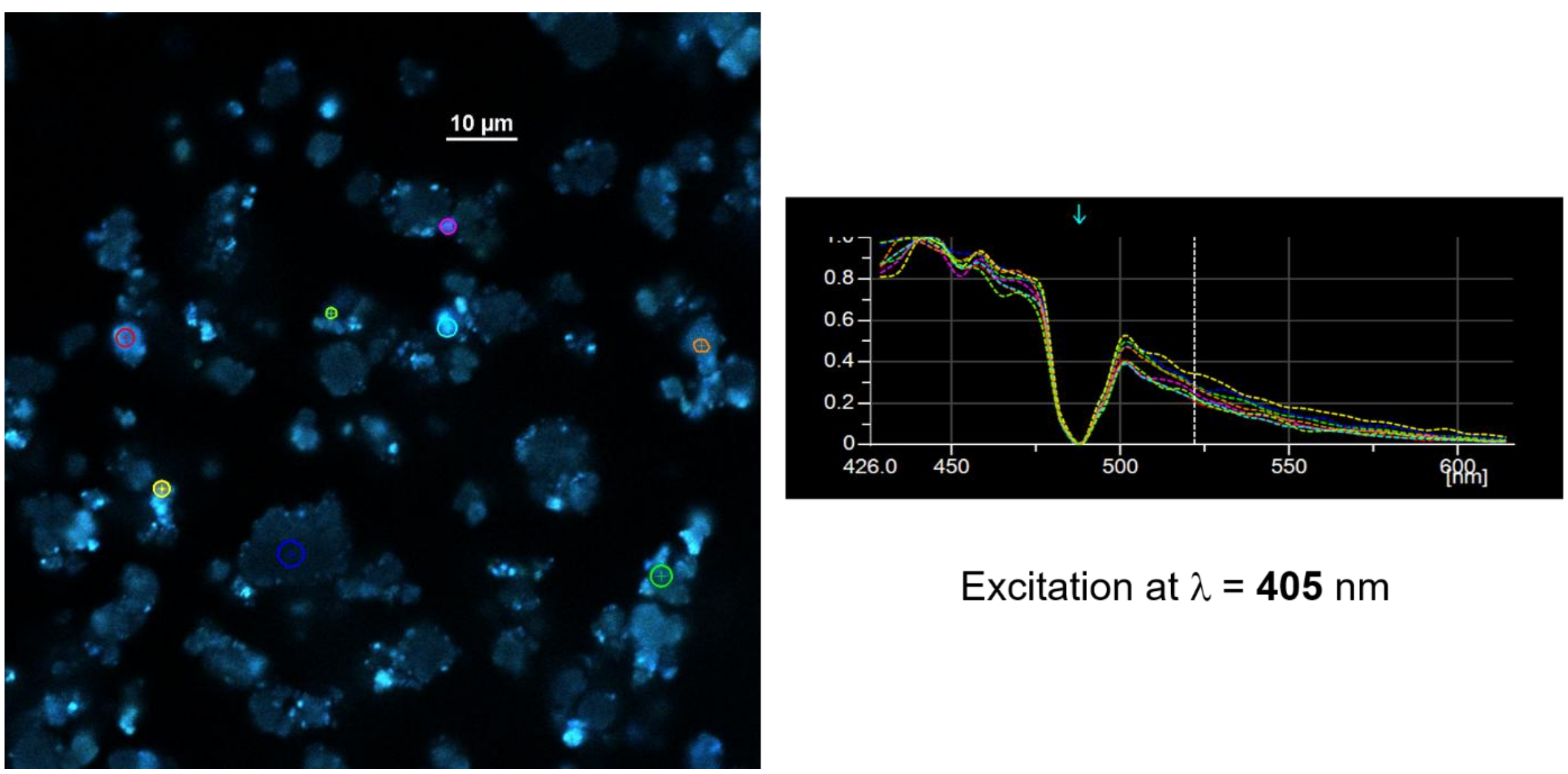

Figure S17. Confocal image (left) and spectra (right) of selected regions of interest in the UiO-68 powder sample for excitation at λ = 405 nm.

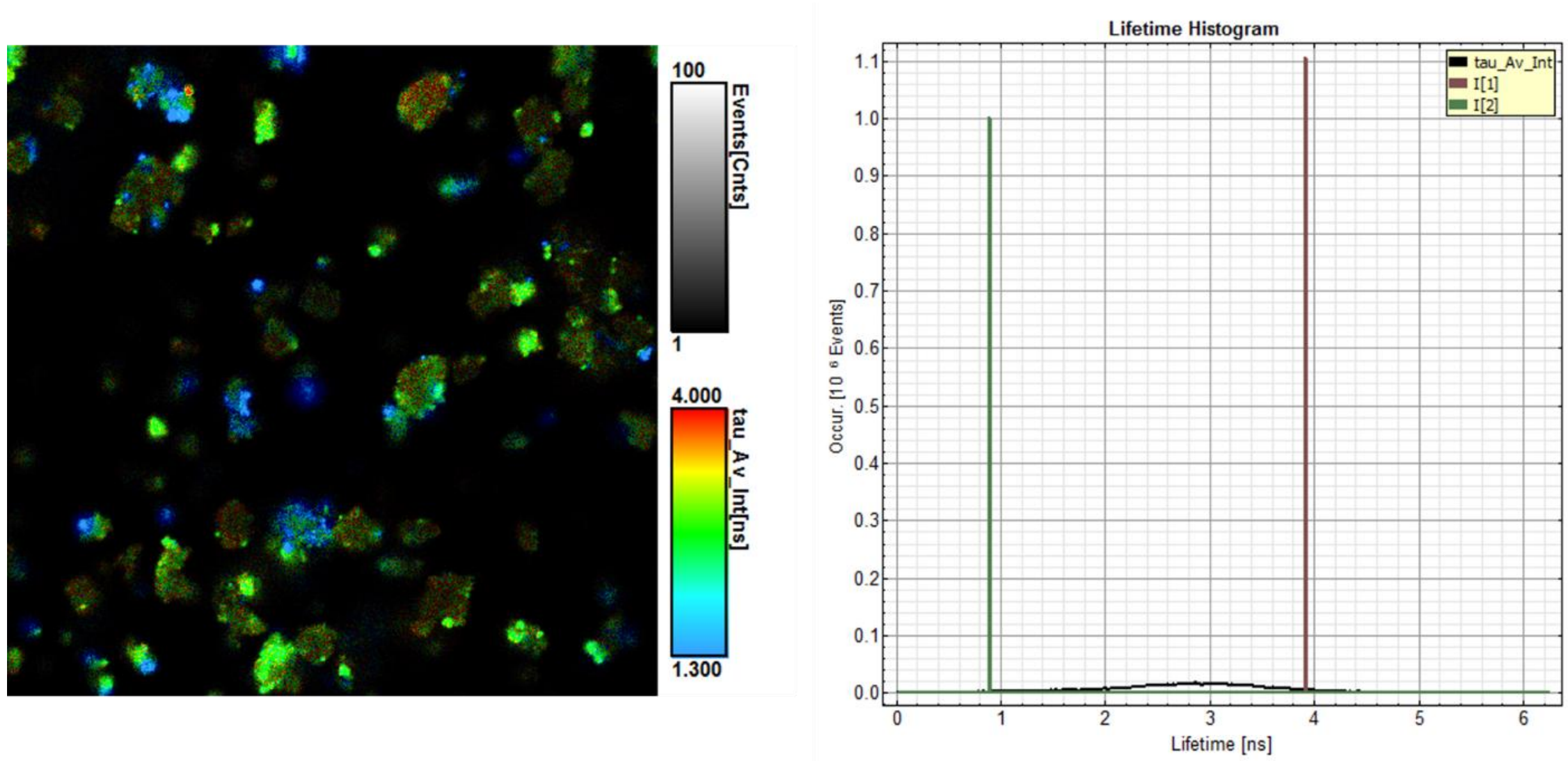

Figure S18. Fluorescence lifetime image of UiO-68 (left) and histogram indicating the frequency of the lifetimes as well as the average lifetime in the fitted image (right) (excitation at 405 nm and emission measured at 480 nm).

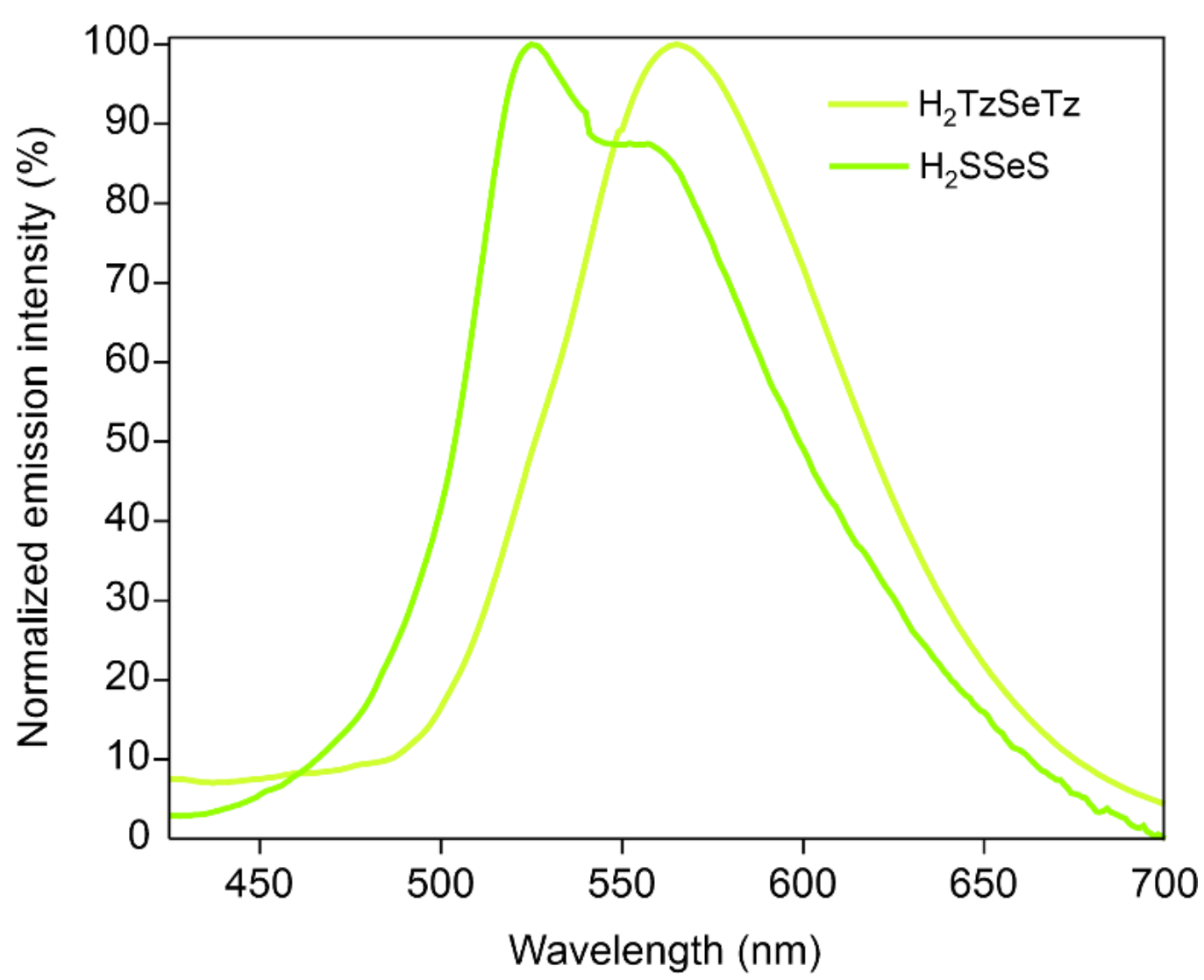

Figure S19. Normalized solid-state emission spectra of H_2SSeS and $H_2TzSeTz$. The colors used for the graphs are the real emission colors: RGB 149/255/0 and 207/255/46 for H_2SSeS and $H_2TzSeTz$, respectively.

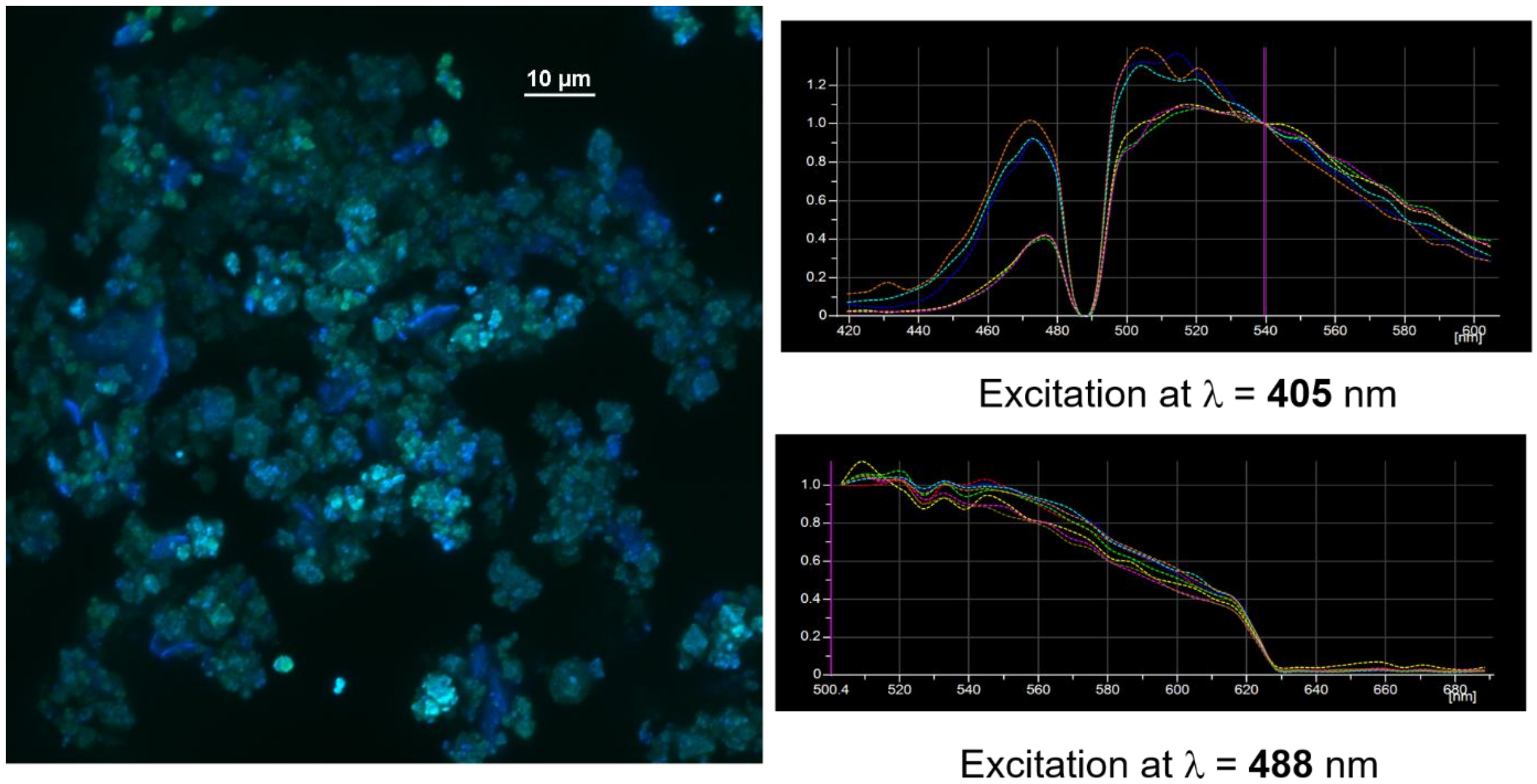

Figure S20. UiO-68-TzSeTz confocal image and spectra.

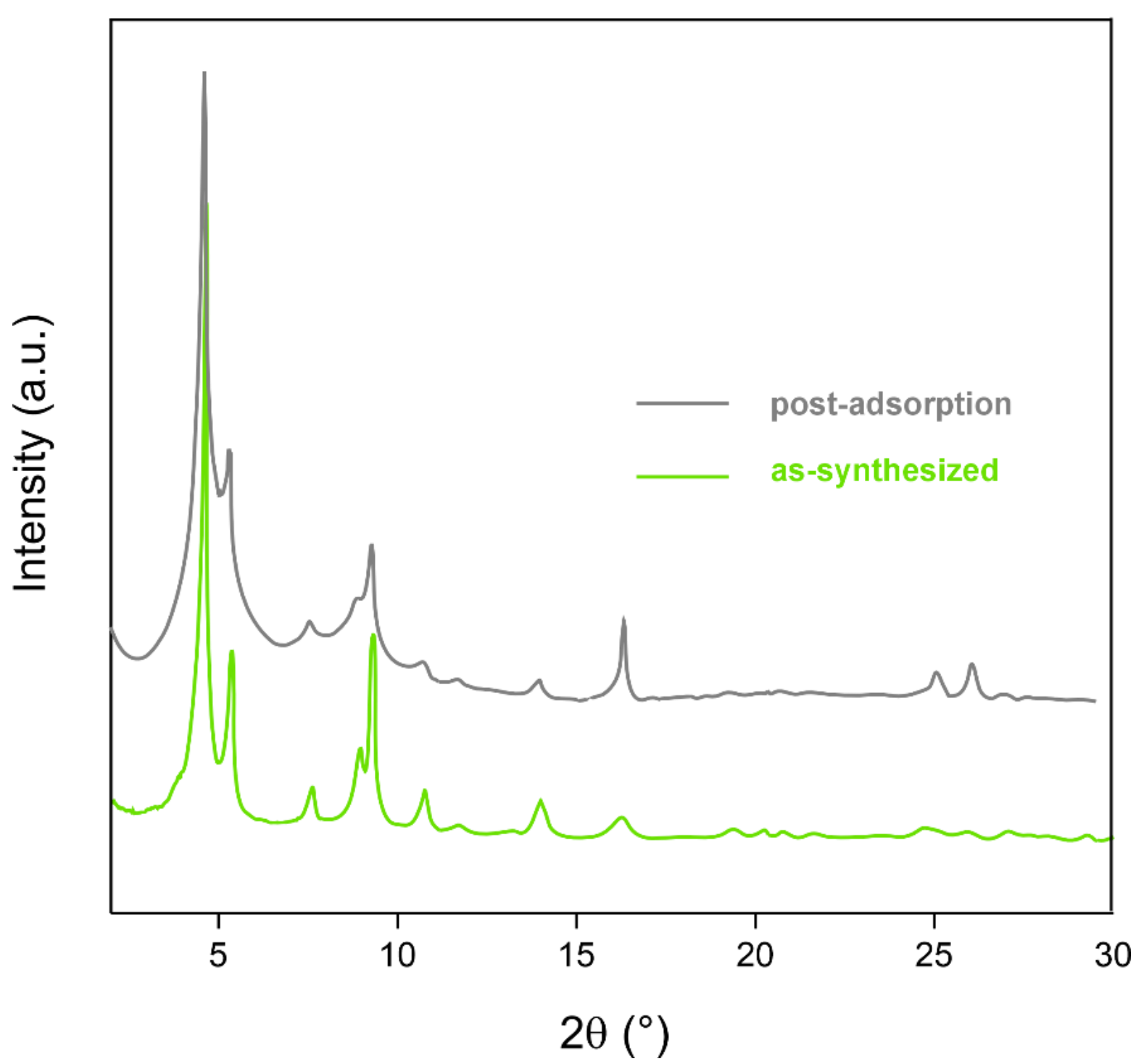

Figure S21. PXRD patterns of **UiO-68-SSeS** before (green) and after (gray) suspension in distilled water for 24 h prior to the adsorption tests.

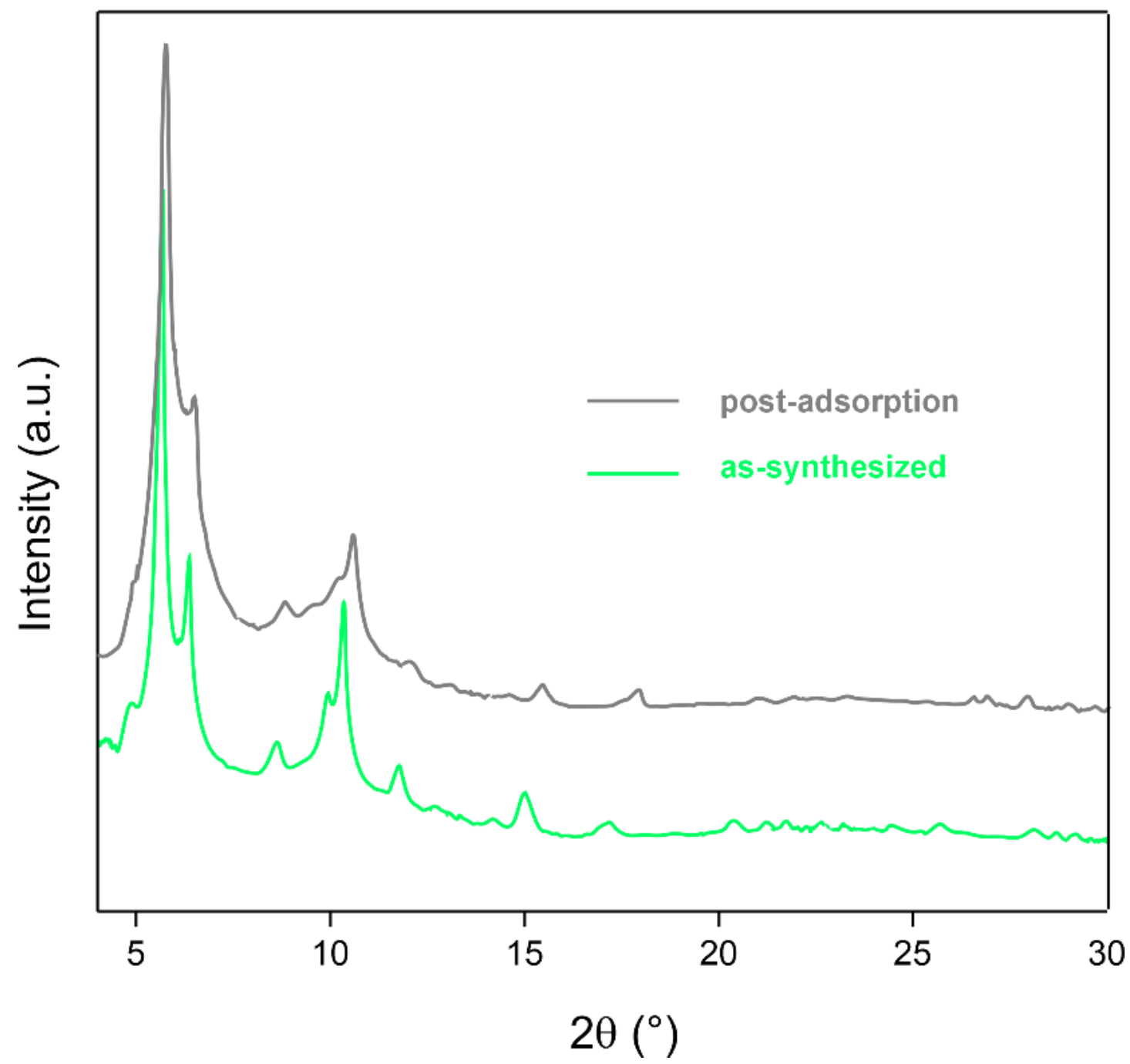

Figure S22. PXRD patterns of **UiO-68-TzSeTz** before (green) and after (gray) suspension in distilled water for 24 h prior to the adsorption tests.

UPLC-MS/MS analysis details

UPLC-MS/MS analyses on PFASs were performed by utilising an UPLC-MS/MS Waters ACQUITY UPLC H-Class PLUS – XEVO TQS Micro mass detector. The chromatographic separation was performed on a reverse-phase Waters Acquity UPLC CSH Phenyl-Hexyl (1.7 µm, 2.1 × 100 mm) column and a Waters Isolator Column (2.1 × 50 mm). The column temperature was set at 307 K, the flow rate at 0.3 mL/min, and the injection volume at 40 µL. The total runtime for PFASs was 21 minutes. The mobile phase consists of a biphasic gradient, with NH_4OAc 2 mM in a mixture of ultrapure water : methanol (95 : 5) as phase A, and NH_4OAc 2 mM in MeOH as phase B. The composition of the mobile phase varies according to the gradient program reported in Table S4 for PFASs analyses. Mass details and the transitions monitored for each analyte are reported in Table S5. The calibration curves were calculated by using the average value of two subsequent UPLC-MS/MS injections. Calibration curve solutions (0.01, 0.05, 0.1, 0.5, 1, 2.5 µg/L) were freshly prepared by diluting the methanolic PFAS stock solution with laboratory phase A and injected before each analytical batch. The regression equations of the calibration curves were found to be linear within the range of 2.5-0.01 or 2.5-0.05 or 2.5-0.1 µg/L, depending on the analyte (Table S5, LOQ). The results reported in the Tables below are expressed as the mean of three replicates ± SD.

Table S4. Elution gradients used for PFAS analyses. Mobile phases: (A) MeOH:aqueous NH_4OAc 2 mM 95:5; (B) NH_4OAc 2 mM in MeOH.

Time (min)	Flow (mL/min)	%A	%B
0.00	0.300	100.0	0.0
1.00	0.300	80.0	20.0
6.00	0.300	55.0	45.0
13.00	0.300	20.0	80.0
15.00	0.350	5.0	95.0
17.00	0.350	5.0	95.0
18.00	0.300	100.0	0.0
21.00	0.300	100.0	0.0

Table S5. LC-MS/MS parameters for all PFAS target analytes using an UPLC-MS/MS Waters ACQUITY UPLC H-Class PLUS – XEVO TQS Micro mass detector.

Name		Monitored transition (ESI^-)	Collision energy (eV)	LOQ (µg/L)
**PFHxS**	Perfluorohexanesulfonic acid	398.96 → 79.90	38	0.01
**PFOA**	Perfluorooctanoic acid	412.98 → 168.98	18	0.01
**PFNA**	Perfluorononanoic acid	462.96 → 218.97	16	0.01
**PFOS**	Perfluoroocatnsulfonic acid	498.90 → 79.90	54	0.01
**PFDA**	Perfluorodecanoic acid	513.12 → 469.00	10	0.01
**PFUnDA**	Perfluoroundecanoic acid	562.96 → 519.06	10	0.01

References

(1) Krause, L.; Herbst-Irmer, R.; Sheldrick, G. M.; Stalke, D. Comparison of Silver and Molybdenum Microfocus X-ray Sources for Single-Crystal Structure Determination. *J. Appl. Crystallogr.* **2015**, *48*, 3–10.

(2) Burla, M. C.; Caliandro, R.; Carrozzini, B.; Cascarano, G. L.; Cuocci, C.; Giacovazzo, C.; Mallamo, M.; Mazzone, A.; Polidori, G. Crystal Structure Determination and Refinement *via* SIR2014. *J. Appl. Cryst.* **2015**, *48*, 306–309.

(3) Sheldrick, G. Crystal Structure Refinement with SHELXL. *Acta Cryst.* **2015**, *C71*, 3–8.

(4) *Mercury 2026.1.1*, Cambridge Crystallographic Data Center, Cambridge, UK.

(5) Deria, P.; Mondloch, J. E.; Tylianakis, E.; Ghosh, P.; Bury, W.; Snurr, R. Q.; Hupp, J. T.; Farha, O. K. Perfluoroalkane Functionalization of NU-1000 via Solvent-Assisted Ligand Incorporation: Synthesis and CO_2 Adsorption Studies. *J. Am. Chem. Soc.* **2013**, *135*, 16801–16804. doi: 10.1021/ja408959g

(6) The empty volume was estimated with the software *Mercury* using a probe radius of 1.2 Å and a grid of pace 0.3 Å; *Mercury 2026.1.1*, Cambridge Crystallographic Data Center, Cambridge, UK.

(7) Manna, K.; Ji, P.; Lin, Z.; Greene, F. X.; Urban, A.; Thacker, N. C.; Lin, W. Chemoselective Single-Site Earth-Abundant Metal Catalysts at Metal–Organic Framework Nodes. *Nat. Commun.* **2016**, *7*, 12610.

(8) Schaate, A.; Roy, P.; Godt, A.; Lippke, J.; Waltz, F.; Wiebcke, M.; Behrens, P. Modulated Synthesis of Zr-Based Metal–Organic Frameworks: From Nano to Single Crystals. *Chem. – Eur. J.* **2011**, *17*, 6643–6651.
